# Resident stroma-secreted chemokine CCL2 governs myeloid-derived suppressor cells in the tumor microenvironment

**DOI:** 10.1172/jci.insight.148960

**Published:** 2022-01-11

**Authors:** May Wathone Oo, Hotaka Kawai, Kiyofumi Takabatake, Shuta Tomida, Takanori Eguchi, Kisho Ono, Qiusheng Shan, Toshiaki Ohara, Saori Yoshida, Haruka Omori, Shintaro Sukegawa, Keisuke Nakano, Kuniaki Okamoto, Akira Sasaki, Hitoshi Nagatsuka

**Affiliations:** 1 Department of Oral Pathology and Medicine, Graduate School of Medicine, Dentistry and Pharmaceutical Sciences, Okayama University, Okayama, Japan.; 2 Center for Comprehensive Genomic Medicine, Okayama University Hospital, Okayama, Japan.; 3 Department of Dental Pharmacology,; 4 Advanced Research Center for Oral and Craniofacial Sciences,; 5 Department of Oral and Maxillofacial Surgery, and; 6 Department of Pathology and Experimental Medicine, Graduate School of Medicine, Dentistry and Pharmaceutical Sciences, Okayama University, Okayama, Japan.; 7 Preliminary Examination Room, Okayama University Hospital, Okayama, Japan.; 8 Department of Oral and Maxillofacial Surgery, Kagawa Prefectural Central Hospital, Kagawa, Japan.

**Keywords:** Oncology, Cytokines, Tumor suppressors

## Abstract

Accumulating evidence has shown that cancer stroma and BM-derived cells (BMDCs) in the tumor microenvironment (TME) play vital roles in tumor progression. However, the mechanism by which oral cancer stroma recruits any particular subset of BMDCs remains largely unknown. Here, we sought to identify the subset of BMDCs that is recruited by cancer stroma. We established a sequential transplantation model in *BALB/c* nude mice, including (a) BM transplantation of GFP-expressing cells and (b) coxenografting of patient-derived stroma (PDS; 2 cases, designated PDS1 and PDS2) with oral cancer cells (HSC-2). As controls, xenografting was performed with HSC-2 alone or in combination with normal human dermal fibroblasts (HDF). PDS1, PDS2, and HDF all promoted BMDC migration in vitro and recruitment in vivo. Multicolor immunofluorescence revealed that the PDS coxenografts recruited Arginase-1^+^CD11b^+^GR1^+^GFP^+^ cells, which are myeloid-derived suppressor cells (MDSCs), to the TME, whereas the HDF coxenograft did not. Screening using microarrays revealed that PDS1 and PDS2 expressed *CCL2* mRNA (encoding C-C motif chemokine ligand 2) at higher levels than did HDF. Indeed, PDS xenografts contained significantly higher proportions of CCL2^+^ stromal cells and CCR2^+^Arginase-1^+^CD11b^+^GR1^+^ MDSCs (as receiver cells) than the HDF coxenograft. Consistently, a CCL2 synthesis inhibitor and a CCR2 antagonist significantly inhibited the PDS-driven migration of BM cells in vitro. Furthermore, i.p. injection of the CCR2 antagonist to the PDS xenograft models significantly reduced the CCR2^+^Arginase-1^+^CD11b^+^GR1^+^ MDSC infiltration to the TME. In conclusion, oral cancer stroma–secreted CCL2 is a key signal for recruiting CCR2^+^ MDSCs from BM to the TME.

## Graphical abstract

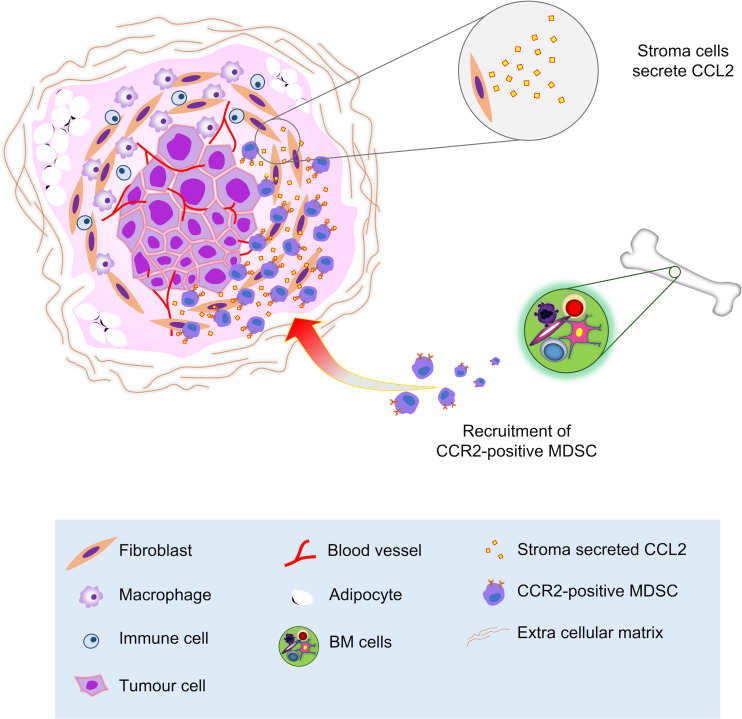

## Introduction

Oral squamous cell carcinoma (OSCC) is one of the most common solid tumors of the head and neck (1, 2). OSCC consists of 2 major components — malignant epithelium and stroma — which make up the tumor microenvironment (TME) (3–5). The stromal components play an active role in supporting tumor cells for progression, invasion, and metastasis (6, 7). Recent studies have revealed that patients who harbor OSCC tumor tissues containing a higher proportion of stromal components show a poorer prognosis (8, 9). The stromal components contain a heterogeneous population of cells that derive from resident stromal cells and recruiting cells, namely cancer-associated fibroblasts (CAFs), tumor-associated endothelial cells (TECs), tumor-associated macrophages (TAMs), and myeloid-derived suppressor cells (MDSCs) (10–12).

MDSCs are BM-derived immature myeloid cells possessing immunosuppressive functions (13, 14). MDSCs emerge and accumulate under chronic inflammatory conditions, including cancers (15). C-C motif chemokine ligand 2 (CCL2), also known as monocytic chemotactic protein (MCP-1), and the CCL2 receptor (CCR2) play crucial roles in MDSC migration. CCL2 is secreted by tumors in prostate cancer, breast cancer, malignant glioma, colorectal cancer (CRC), and lung carcinoma. The CCL2/CCR2 axis has been proposed as a potential target for decreasing MDSCs recruitment and improving cancer treatment (16). However, the role of stroma-secreted CCL2 in MDSC recruitment in OSCC remains unclear.

In the present study, we sought new insights into the interconnection between resident stroma and MDSCs in the TME. To this end, we established a potentially novel patient-derived stroma xenograft (PDSX) model. The PDSX model permits an in vivo recreation of the TME to reproduce the interaction between the resident stroma and MDSCs, generating a system resembling the human cancer–TME interaction under natural growth conditions. We established the PDSX model by coxenografting nude mice with the human OSCC cell line (HSC-2) together with patient-derived stromal (PDS) cells (designated PDS1 and PDS2) isolated from 2 OSCC patients. As a control, we established a coxenograft model of a normal human dermal fibroblast (HDF) cell line with HSC-2 cells. As another control for this model, we xenografted recipient mice with HSC-2 alone. Using these animal models, we investigated (a) whether the resident stroma alters the infiltration of BMDCs into the TME; (b) whether the resident stroma alters particular subsets of BMDCs, including MDSCs; and (c) the gene expression signature of stromal cells, a profile that is expected to be essential for the migration of MDSCs. Our data provide insight showing that MDSC recruitment to the TME depends on the secretion by the resident stroma of CCL2, a chemokine ligand of the receptor CCR2 expressed in MDSCs.

## Results

### Isolation and characterization of PDS.

We first performed the primary culture of surgical operative OSCC tissue specimens of 2 patients suffering from OSCC in the tongue. Then we separated the PDS cells from tumor cells based on the different adherent abilities to culture plate of stromal and tumor cells; OSCC tumor cells have a stronger adherent ability than stromal cells. We dissociated the primary cultured cells from the culture dishes by Accutase. The early dissociated cells were isolated and cultured again. After isolation, we confirmed the isolation of stromal cells by analyzing the expression of a mesenchymal marker, vimentin, and an epithelial marker, E-cadherin. We used human OSCC cell line (HSC-2) and primarily cultured normal HDF as positive control cells that express E-cadherin and vimentin, respectively. PDS1, PDS2, and HDF (the positive control) expressed vimentin but not E-cadherin (Supplemental Figure 1A; supplemental material available online with this article; https://doi.org/10.1172/jci.insight.148960DS1). On the other hand, the expression of E-cadherin was observed in HSC-2, but vimentin was undetectable, as expected for this tumor type (17, 18). Furthermore, we evaluated whether the isolated stroma has the specific character of cancer-associated cells such as CAFs, by detecting fibroblast activation protein (FAP), a known marker expressed in CAFs (19). We evaluated the expression of FAP by Western blotting and observed FAP expression in PDS1 and PDS2 and slight expression of FAP in HDF (Supplemental Figure 1A). In contrast, FAP was undetectable in HSC-2. In addition, PDS1 and PDS2 were morphologically similar to HDF, exhibiting a spindle-shaped or stellate-like morphology with network-like intercellular connections (Supplemental Figure 1B).

These data indicate that the isolated PDS cells consisted primarily of mesenchymal cells with the character of cancer-associated fibroblasts.

### Stromal cells promote the migration of BM cells.

**Figure 1:**
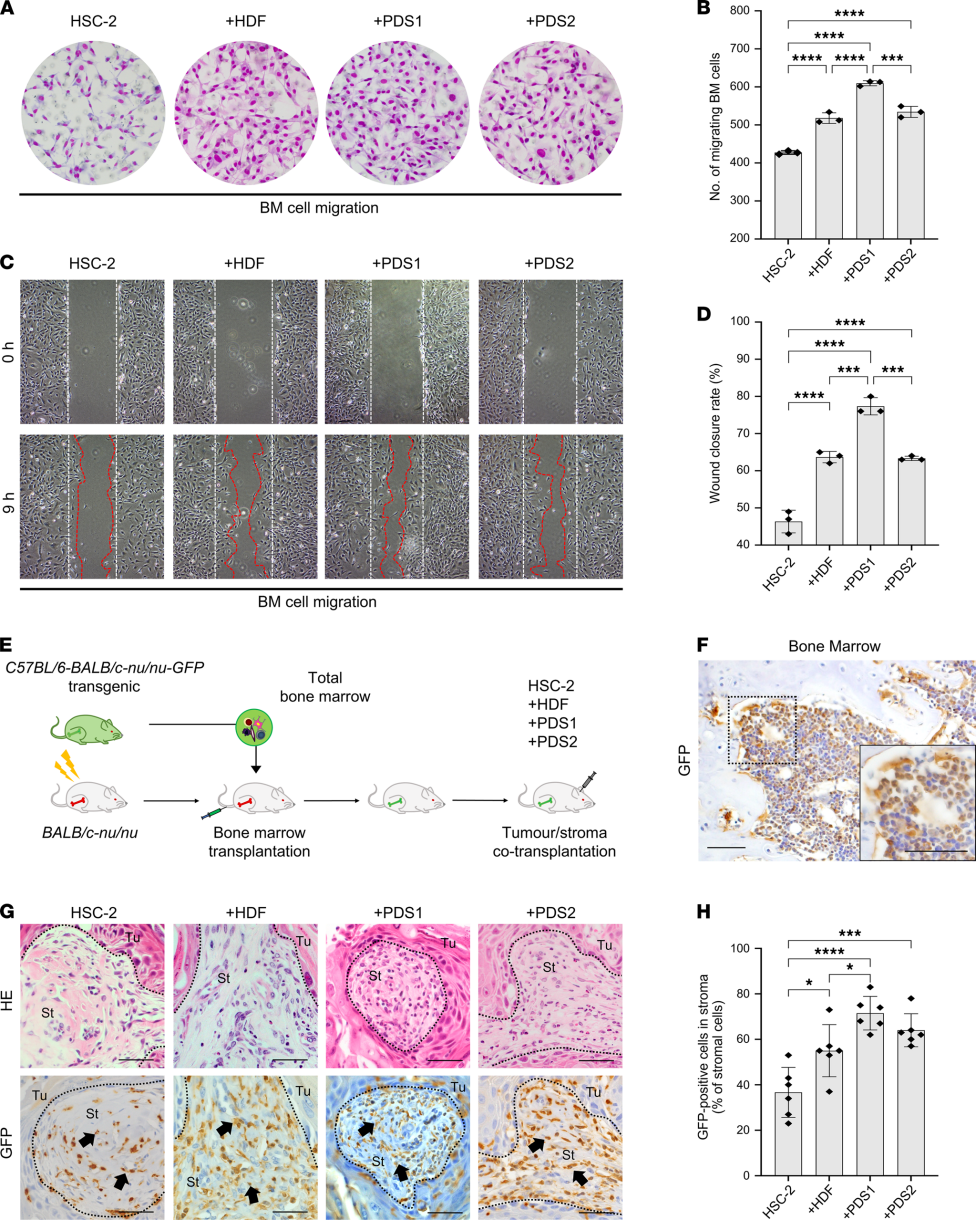
Stroma influences BMDCs infiltration into the tumor microenvironment. (**A**) Representative images of Transwell migration. We allowed the migration of BM cells from the upper chamber to the lower chamber of different tumor/stroma cocultures. HSC-2, HSC-2–alone culture; +HDF, HSC-2 + HDF coculture; +PDS1, HSC-2 + PDS1 coculture; +PDS2, HSC-2 + PDS2 coculture. (**B**) The number of migrating BM cells per field (5 fields per group, *n* = 3). (**C**) Representative images of wound closure assay. We allowed the migration of BM cells in the different conditioned media. White dotted line, wounded area at 0 hours (h). Red dotted line, leading edge of migrating cells in 9 h. (**D**) Wound closure rate (%) of BM cells at 9 h after wound (5 fields per group, *n* = 3). (**E**) Illustration of BM transplantation (BMT) and tumor/stroma coxenograft models. The whole-body irradiated nude mice were subjected to BMT of GFP^+^ BM cells via tail veins. Tumor/stroma was transplanted s.c. to the head 4 weeks after the BMT. Four weeks later, tumors and bones were harvested for analysis of GFP^+^ cell distribution. (**F**) IHC of GFP on BM of BMT mouse. An image enclosed in a rectangle on the bottom right is a higher magnified image. Scale bars: 50 μm. (**G**) BMDCs infiltration into tumor stroma in tumor xenograft models. Upper panels, H&E staining. Lower panels, IHC of GFP. Arrows indicate positive stromal cells. Dotted lines represent the boundary of the tumor (Tu) and stroma (St) area. Scale bars: 100 μm. HSC-2, HSC-2–alone xenograft (**H**) The rate of infiltrating BMDCs into the tumor stroma (% of stromal cells). +HDF, HSC-2 + HDF coxenograft; +PDS1, HSC-2 + PDS1 coxenograft; +PDS2, HSC-2 + PDS2 coxenograft (5 fields per mouse, *n* = 6). All data are shown as mean ± SD. Statistical analyses were performed using 1-way ANOVA followed by Tukey’s multiple-comparison post hoc test; *P < 0.05, ***P < 0.001, ****P < 0.0001.

To evaluate whether the tumor-stroma interaction alters BMDC migration in vitro, we next performed a Transwell migration assay and wound closure assay. We seeded HSC-2 with or without stromal cells in the lower chamber of Transwells and allowed BM cells to migrate from the upper chamber. Stromal cells (PDS1, PDS2, and HDF) cocultured with HSC-2 cancer cells significantly promoted higher BM cell migration (610, 534, and 518 cells per 1 field, respectively), compared with the HSC-2–alone culture (427 cells per 1 field) (Figure 1, A and B). For the wound closure assay, we examined whether the conditioned media from HSC-2–alone culture or the coculturing (HSC-2 + HDF, HSC-2 + PDS1, or HSC-2 + PDS2) altered the migratory activity of BM cells. We collected the conditioned media from tumor-alone culture or different tumor/stroma cocultures and allowed the BM cells to migrate in different conditioned media. The tumor/stroma coculture–derived conditioned media from HSC-2 + PDS1, HSC-2 + PDS2, and HSC-2 + HDF promoted more rapid wound closure by BM cells at the rate of 77%, 63%, and 64%, respectively, compared with the conditioned medium from the HSC-2 alone (46%) (Figure 1, C and D).

These results indicate that a putative factor released upon the stromal cells’ interaction with cancer cells is important for stimulating BM cell migration.

### Tumor-stroma interaction induces BMDC recruitment into the TME.

To investigate whether resident stroma can provide a favorable environment for BMDCs recruitment into the TME, we next analyzed BMDC infiltration into the TME in the coxenograft model of the 3 different stromal cell types with HSC-2 cancer cells. We established a BM transplantation (BMT) mouse model bearing a tumor/stroma complex (Figure 1E). To trace BMDCs, we allografted GFP^+^ BM cells into GFP^–^ nude mice (via BMT) and tracked the GFP^+^ BM cells in these animals. We next confirmed the establishment of BMT by IHC staining for GFP in femoral BM. All BMT mice demonstrated the full replacement of BM with GFP^+^ cells (Figure 1F). We next coxenografted stromal cells with HSC-2 cancer cells (HSC-2 + HDF, HSC-2 + PDS1, HSC-2 + PDS2, or HSC-2 alone) into the BMT mice. GFP^+^ BMDCs were recruited into all tumor xenograft models 4 weeks after the transplantation (Figure 1G). GFP^+^ cells were localized primarily in the stromal area rather than in the tumor area. The GFP^+^ BMDCs were rounded, dendritic, or spindle shaped in morphology. We next examined whether stroma interacting with the tumors altered BMDC recruitment efficiency by analyzing the rate of GFP^+^ cell infiltration into the stromal area (Figure 1G). The cotransplanted mice (HSC-2 + PDS1, HSC-2 + PDS2, and HSC-2 + HDF) showed significantly higher GFP^+^ cells infiltration (72%, 64%, and 55%, respectively) into the stromal area than the HSC-2–alone transplanted mice (37%) (Figure 1H). These data are consistent with the in vitro studies of BM cell migration described above and, thus, indicate that BMDC recruitment into the TME differed according to the stromal cells.

### PDS cells promote MDSC recruitment into TME.

To determine whether any specific subset of BMDCs was recruited by resident stroma interacting with the tumors, we next immunostained tumor tissues using a panel of antibodies that recognize cellular subsets in the TME, including αSMA as a marker of CAFs (20, 21), CD34 as a marker of TECs (12, 22, 23), CD11b as a marker of TAMs (24–26), and coexpression of CD11b and GR1 as a marker of MDSCs (27). To examine whether these subsets were equivalent to BM-derived GFP^+^ cells, we analyzed the coexpression of GFP with these markers among the xenograft groups. We found αSMA^+^ spindle cells in the stromal area of all tumor models. However, αSMA^+^ cells did not coexpress GFP (Supplemental Figure 2A), suggesting that αSMA^+^ CAFs are not derived from BM. CD34^+^ cells formed the vessel loop structures in all tumor models (Supplemental Figure 2B). However, these cells did not express GFP, suggesting that CD34^+^ TECs are also not derived from BM. CD11b^+^ cells were abundantly found in the stromal and tumor areas in all xenograft models, assuming rounded or dendritic morphologies (Supplemental Figure 2C). We found CD11b^+^GFP^+^ cells abundantly in the stroma of all the tumor models (Supplemental Figure 2D). Indeed, approximately 50% of GFP^+^ cells were CD11b^+^. The rates of CD11b^+^GFP^+^ TAMs in coxenograft models tended to be lower than that in the model injected with HSC-2 alone. These data indicate that BM-derived recruitment of TAMs may be inhibited by stroma-derived factors.

**Figure 2:**
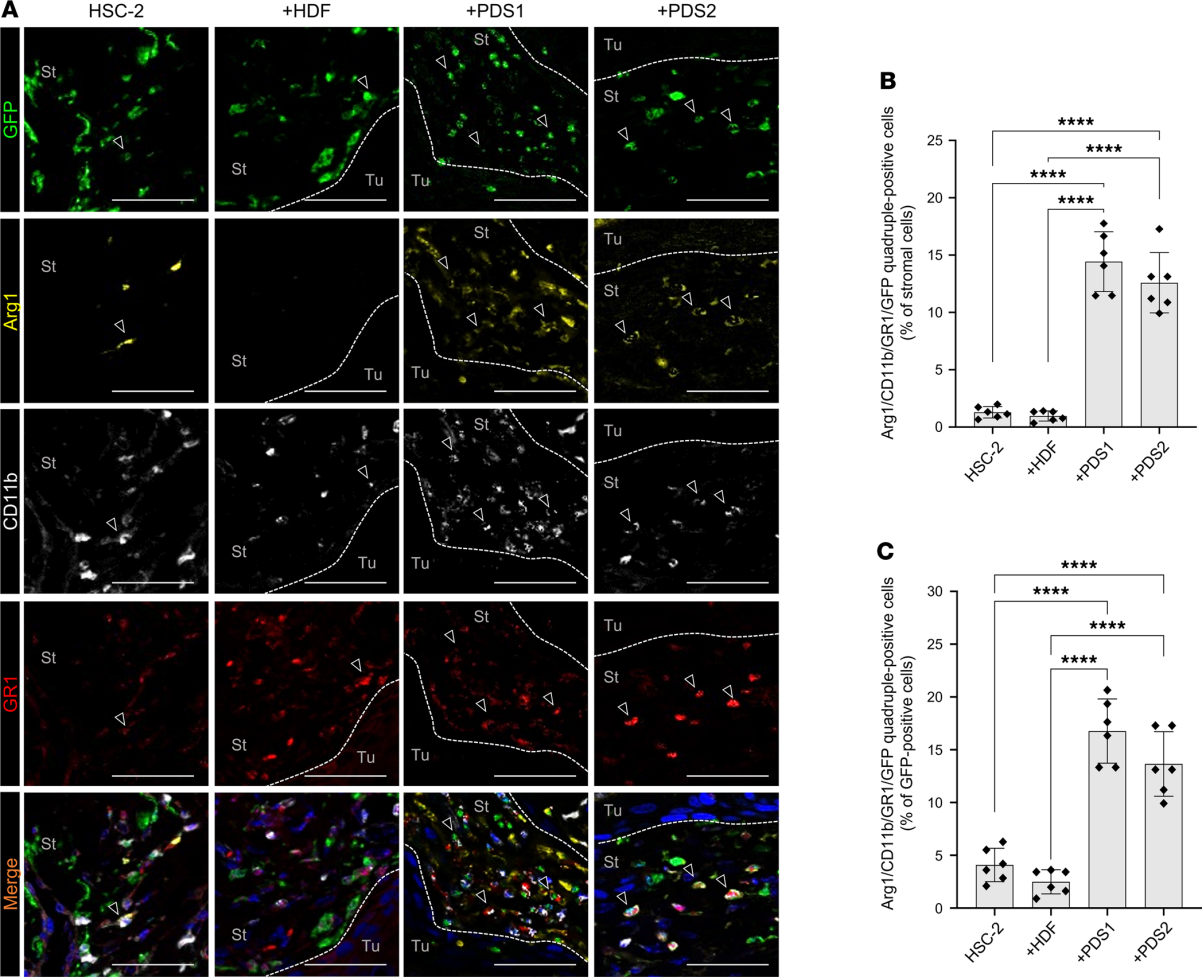
Patient-derived stromal cells promote MDSC recruitment into the TME. MDSCs were analyzed by multicolor IHC on Arg1/CD11b/GR1/GFP. (**A**) Representative images of multicolor IHC detecting GFP (green), Arg1 (yellow), CD11b (white), and GR1 (red). Arrowheads indicate positive stromal cells. Dotted lines represent the boundary of the tumor (Tu) and stroma (St) area. Nuclei are stained with DAPI. Scale bars: 50 μm. (**B**) The rate of Arg1^+^CD11b^+^GR1^+^GFP^+^ cells (% of stromal cells). (**C**) The rate of Arg1^+^CD11b^+^GR1^+^GFP^+^ cells (% of GFP^+^ cells). HSC-2, HSC-2–alone xenograft; +HDF, HSC-2 + HDF coxenograft; +PDS1, HSC-2 + PDS1 coxenograft; +PDS2, HSC-2 + PDS2 coxenograft (5 fields per mouse, *n* = 6). All data are shown as mean ± SD. Statistical analyses were performed using 1-way ANOVA followed by Tukey’s multiple-comparison post hoc test; ****P < 0.0001.

Murine MDSCs are known to express CD11b and GR1 and to provide immunosuppression by producing arginase-1 (Arg1) (27, 28). Therefore, we next identified MDSCs by performing multicolor fluorescence detection on Arg1^+^ cells that exhibited costaining with GFP, CD11b, and GR1. Notably, Arg1^+^ cells appeared to accumulate only in PDSX (HSC-2 + PDS1 and HSC-2 + PDS2) model mice and not in HSC-2 + HDF coxenograft animals (Figure 2A). Arg1^+^CD11b^+^GR1^+^GFP^+^ MDSCs accumulated abundantly in the stromal area of the PDSX models (14% in HSC-2 + PDS1 and 13% in HSC-2 + PDS2), while only a small number of such cells (around 1%) were found in the stromal areas of the HSC-2–alone and HSC-2 + HDF mice (Figure 2B). Moreover, the proportions of Arg1^+^CD11b^+^GR1^+^ MDSCs per GFP^+^ cells were significantly higher in PDSX mice (17% in HSC-2 + PDS1 and 13% in HSC-2 + PDS2) than in HSC-2-alone (1%) and HSC-2 + HDF mice (2%) (Figure 2C).

These data indicate that PDS promotes MDSC infiltration into the TME, although this accumulation was not seen for CAFs, TECs, or TAMs.

### Cellular chemotaxis factor CCL2 is highly expressed in PDS cells.

**Figure 3:**
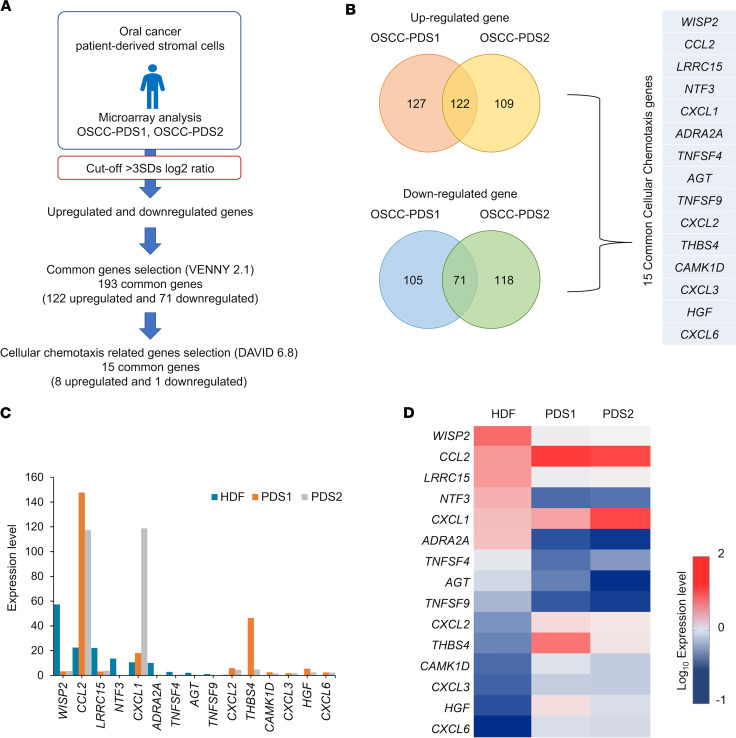
Comprehensive analysis of cellular chemotaxis–related genes revealed that *CCL2* mRNA was highly expressed in the patient-derived stroma. (**A**) Flow chart of cellular chemotaxis–related genes screening. We analyzed the differentially expressed genes (DEGs) of oral squamous cell carcinoma (OSCC) PDS1 and PDS2 compared with HDF with the cut-off of more than 3 times of SD. We selected the common DEGs by Venny 2.1 and observed the cellular chemotaxis–related genes by DAVID bioinformatics Resources 6.8. (**B**) Venn diagram for commonly upregulated and downregulated gene expression among OSCC-PDS1 and OSCC-PDS2, and list of 15 cellular chemotaxis–related genes shared by PDS1 and PDS2. (**C**) Relative mRNA expression levels of 15 cellular chemotaxis genes in HDF, PDS1, and PDS2. (**D**) Heatmap presentation of the expression level of cellular chemotaxis genes in HDF, PDS1, and PDS2.

To clarify the underlying molecular mechanism by which coxenografted stromal cells (PDS1 and PDS2) promoted MDSCs recruitment, we next performed microarray analysis to identify the differentially expressed genes (DEGs) in PDS1 and PDS2 compared with HDF (Figure 3A). We found that 249 genes exhibited higher (by more than 3 SDs) expression levels in PDS1 compared with that in HDF, while 231 genes had higher expression in PDS2 compared with that in HDF; 122 genes were upregulated in common between PDS1 and PDS2 (compared with HDF) (Supplemental Tables 1 and 2 and Figure 3B). Simultaneously, 176 genes were downregulated (to more than 3 SDs) in PDS1 compared with HDF, while 189 genes were downregulated in PDS2 compared with HDF; 71 genes were downregulated in common between PDS1 and PDS2 (compared with HDF) (Supplemental Tables 3 and 4 and Figure 3B). Furthermore, we identified the biological process of common upregulated and downregulated genes of PDS1 and PDS2 using DAVID and screened the cellular chemotaxis–related genes (Supplemental Figure 3). We observed that 15 common genes are relating to cellular chemotaxis in PDS1 and PDS2 (Figure 3B). Among these 15 genes, *CCL2* mRNA expression levels were markedly higher in the PDS1 and PDS2 (Figure 3, C and D).

As for the validation data set, we next confirmed whether the significant DEGs in PDS1 and -2 shared with those in the stroma isolated from other cancer. It has been shown that OSCC and CRC have a common gene expression profile and therapeutic target (29, 30). Therefore, we additionally analyzed the publicly available expression profile of PDS of CRC, for which the isolation method of stromal cells was used similarly to our method. We selected the gene expression profile of isolated PDS from a CRC patient (GSE93253) from the Gene Expression Omnibus GEO database (https://www.ncbi.nlm.nih.gov/geo/), for which the gene expression profile of normal fibroblast (NF) (*n* = 4) versus CRC-PDS (*n* = 3) were evaluated (31). To selectively focus on altered gene expression that was shared between OSCC stroma and CRC stroma, we next compared our OSCC-PDS gene expression data with the CRC-PDS gene expression data; both data sets included relative expression values compared with expression data from normal fibroblasts (Supplemental Figure 4A). Integration of these 3 data sets (OSCC-PDS1, OSCC-PDS2, and CRC-PDS) revealed 33 genes that were upregulated in common and 8 genes that were downregulated in common, providing a total of 41 shared DEGs (Supplemental Figure 4, A–C). Among these 41 DEGs, we identified 9 genes associated with cellular chemotaxis, as assessed by functional annotation clustering and pathway analysis (Supplemental Figure 4, D and E). Notably, this group of 9 genes also included *CCL2,* suggesting that stroma-secreted CCL2 is the important stromal factor in the TME in cancer patients.

### Stroma-secreted CCL2 is the key factor to recruit BMDCs to the TME.

**Figure 4:**
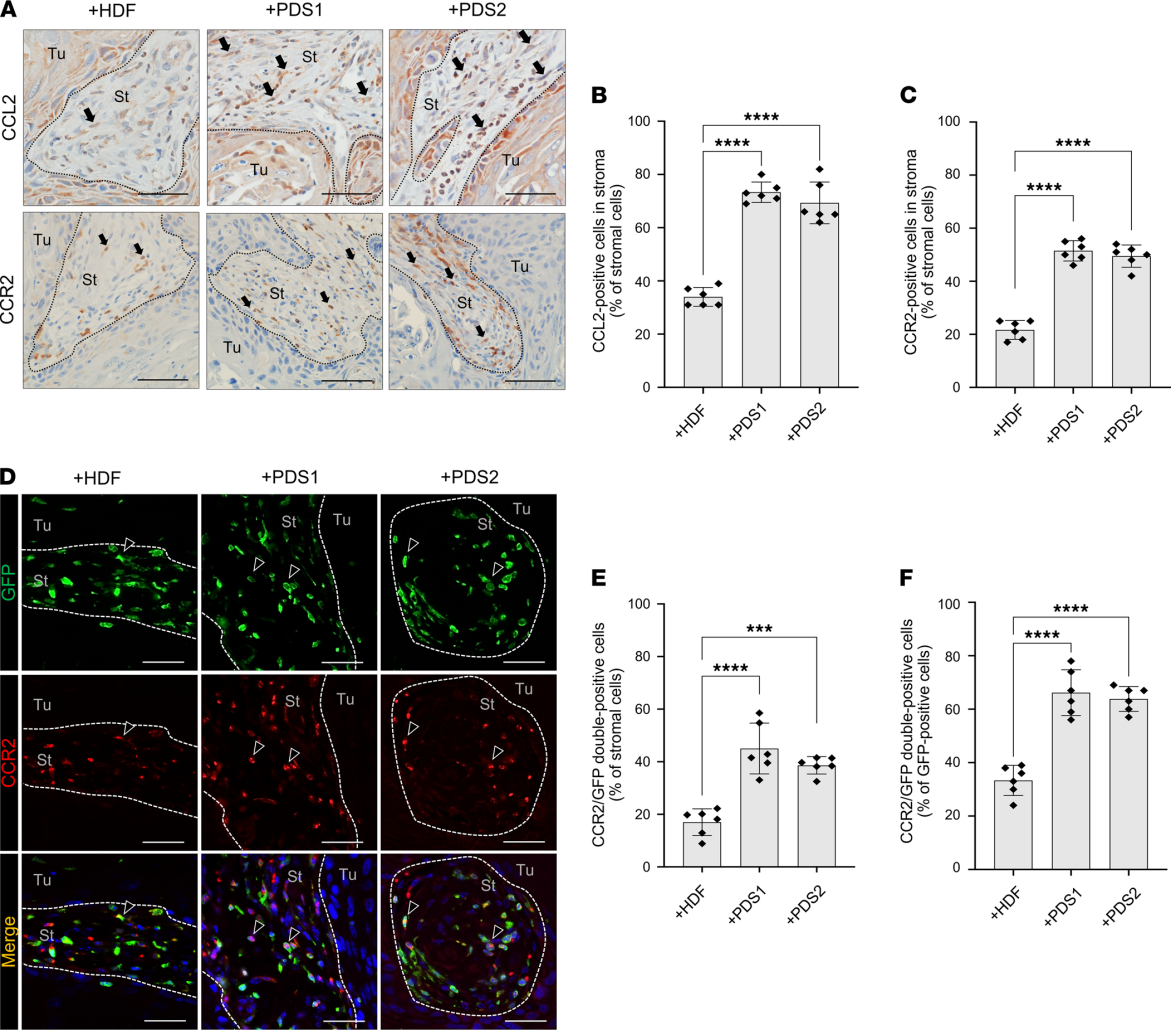
IHC of CCL2^+^ stroma cells and recruited CCR2^+^ BMDCs in TME. (**A**) IHC of CCL2 (upper panels) and CCR2 (lower panels). Arrows indicate CCL2 or CCR2^+^ stromal cells. Dotted lines represent the boundary of the tumor (Tu) and stroma (St) area. Scale bars: 50 μm. (**B**) The rate of CCL2^+^ cells in the stroma (% of stromal cells). (**C**) The rate of CCR2^+^ cells in the stroma (% of stromal cells). (**D**) Representative images of double-fluorescent IHC for GFP (green) and CCR2 (red). Nuclei are stained with DAPI. Arrowheads indicate positive stromal cells. Dotted lines represent the boundary of the tumor (Tu) and stroma (St) area. Scale bars: 50 μm. (**E**) The rate of CCR2^+^GFP^+^ cells (% of stromal cells). (**F**) The rate of CCR2^+^GFP^+^ cells (% of GFP^+^ cells). +HDF, HSC-2 + HDF coxenograft; +PDS1, HSC-2 + PDS1 coxenograft; +PDS2, HSC-2 + PDS2 coxenograft (5 fields per mouse, *n* = 6). All data are shown as mean ± SD. Statistical analyses were performed using 1-way ANOVA followed by Tukey’s multiple-comparison post hoc test; ***P < 0.001, ****P < 0.0001.

Next, we examined CCL2 protein expression in tumor/stroma coxenografted tissues. We detected spindle-shaped CCL2^+^ fibroblast-like cells in the stromal area (Figure 4A). Notably, the rates of CCL2^+^ cells in the stroma of PDSX (73% in HSC-2 + PDS1 and 69% in HSC-2 + PDS2) were significantly higher than those in the HSC-2 + HDF coxenograft (34%), consistent with the microarray data (Figure 4B). CCR2 has been reported to serve as the primary functional receptor for CCL2 in the TME, and the CCL2/CCR2 axis is known to play a crucial role in tumor progression (32). Therefore, we next examined whether PDS recruited CCR2^+^GFP^+^ BM-derived cells to the TME in the coxenograft models. CCR2^+^ cells were localized primarily in the stromal area and exhibited round-shaped morphologies, like those seen for MDSCs, in all coxenografts (Figure 4A). However, the CCR2^+^ cells infiltrated into the stroma of PDSX (52% in HSC-2 + PDS1 and 50% in HSC-2 + PDS2) to a significantly higher degree compared with the HSC-2 + HDF coxenograft (22%) (Figure 4C). In addition, these CCR2^+^ cells expressed GFP (Figure 4D). CCR2^+^GFP^+^ cells showed stronger infiltration into the stroma in PDSX (45% in HSC-2 + PDS1 and 39% in HSC-2 + PDS2) than in the HSC-2 + HDF model (17%) (Figure 4E). The rate of CCR2^+^GFP^+^ cells per total GFP^+^ BM cells was significantly higher in the PDSX (66% in HSC-2 + PDS1 and 63% in HSC-2 + PDS2) than in the HSC-2 + HDF model (33%) (Figure 4F).

**Figure 5:**
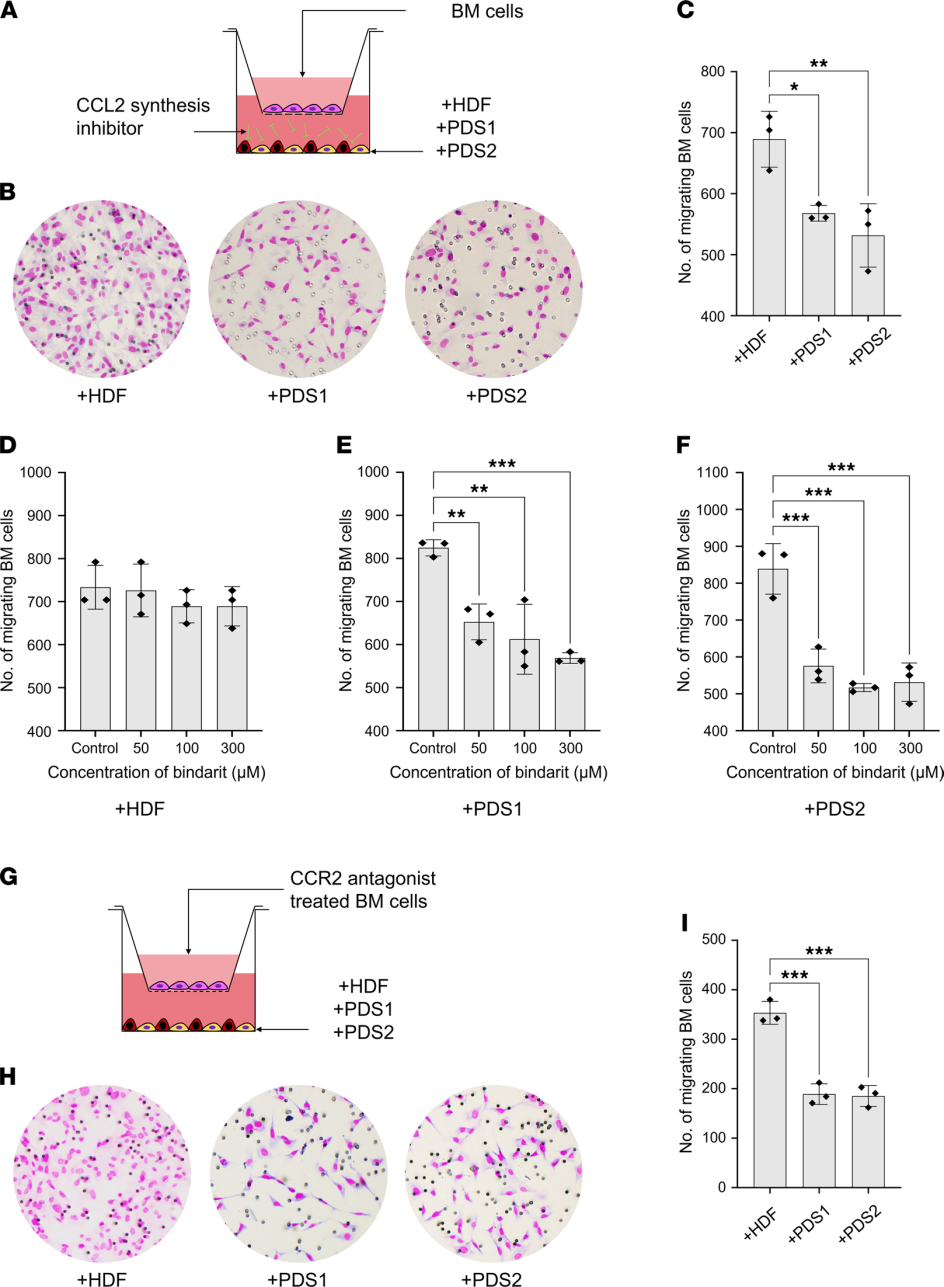
The inhibition of the CCL2/CCR2 axis significantly lowered stroma-driven BM cell migration. (**A**) Illustration of Transwell migration assessment of BM cells in the presence of CCL2 inhibitor bindarit (shown as the green bars). CCL2 inhibition with 50 μM (0.1% DMSO), 100 μM (0.2% DMSO), and 300 μM (0.6% DMSO) of bindarit was performed for 4 h before migration. As control 0.6% DMSO was used. (**B**) Representative images of Transwell migration of BM cells in the presence of bindarit. (**C**) The number of migrating BM cells among the different tumor/stroma cocultures in the presence of bindarit (300 μM). (**D**–**F**) The comparison of BM cell migration in different concentrations of bindarit in +HDF, +PDS1, and +PDS2. (**G**) Illustration of Transwell migration assessment of BM cells pretreated with CCR2 antagonist RS 102895 (20 μM) for 1 h. (**H**) Representative images of Transwell migration of BM cells pretreated with RS 102895. (**I**) The number of BM cells migrating after the treatment with RS 102895 (20 μM) in the different tumor/stroma cocultures. +HDF, HSC-2 + HDF coculture; +PDS1, HSC-2 + PDS1 coculture; +PDS2, HSC-2 + PDS2 coculture (5 fields per group, *n* = 3). All data are shown as mean ± SD. Statistical analyses were performed using Student’s *t* test for comparison of 2 groups and 1-way ANOVA followed by Tukey’s multiple-comparison post hoc test for comparison of more than 2 groups; *P < 0.05 **P < 0.01, ***P < 0.001.

We next tested whether the inhibition of CCL2 synthesis in tumor/stroma coculture altered BM cell migration. We assessed BM cell migration in the presence or absence of a CCL2 production inhibitor, bindarit, in tumor/stroma cocultures (HSC-2 + HDF, HSC-2 + PDS1, and HSC-2 + PDS2) (Figure 5A). We found that CCL2 inhibition effectively decreased BM cell migration in the HSC-2 + PDS1/2 groups (689, 568, and 532 cells per field in HDF coculture, PDS1 coculture, and PDS2 coculture, respectively) (Figure 5, B and C). Moreover, the HSC-2 + PDS cocultures demonstrated significantly slower and dose-dependent BM cell migration, while HSC-2 + HDF showed a similar migration of BM cells at various bindarit concentrations (Figure 5, D–F).

Furthermore, we evaluated whether the inhibition of CCL2 functional receptor CCR2 on the surface of BM cells could alter stroma-driven BM cell migration. Firstly, we pretreated the BM cells with a CCR2 antagonist RS 102895 and evaluated BM cell migration to the tumor/stroma cocultures (HSC-2 + HDF, HSC-2 + PDS1, and HSC-2 + PDS2) (Figure 5G). The data show significantly lower BM migration in HSC-2 + PDS groups (189 cells per field in HSC-2 + PDS1 and 185 cells per field in HSC-2 + PDS2) compared with HSC-2 + HDF (353 cells per field) (Figure 5, H and I). These findings suggest that stroma-secreted CCL2 promoted CCR2^+^ BM cells migration via CCL2/CCR2 axis.

It has been reported that stromal cell–derived factor 1 (SDF-1) and hepatocyte growth factor (HGF) play a vital role in the migration of BM cells to the injured sites (33). Therefore, we also examined whether HGF and SDF-1 were expressed in the stroma. In microarray data, *SDF1* mRNA was not included among the shared DEGs assessed for a role in cellular chemotaxis, while *HGF* mRNA is expressed at a low level (Figure 3, C and D). Similarly, in tissue analysis using IHC, only a few numbers of the spindle-shaped stromal cells expressed SDF-1 and HGF (Supplemental Figure 5A), and there was no significant difference in the SDF-1 (approximately 4% of stromal cells) and HGF (approximately 15% of stromal cells) expression among the coxenografted stroma (Supplemental Figure 5, B and C). BM-derived GFP^+^ cells did not coexpress CXCR4, the functional receptor for SDF-1, nor cMet, a receptor for HGF (Supplemental Figure 5D). These data suggest that PDS-secreted SDF-1 and HGF might participate less in BMDC recruitment in OSCC TME than in the CCL2/CCR2 axis.

### Stroma-derived chemokine CCL2 recruits CCR2^+^ MDSCs into the TME.

**Figure 6:**
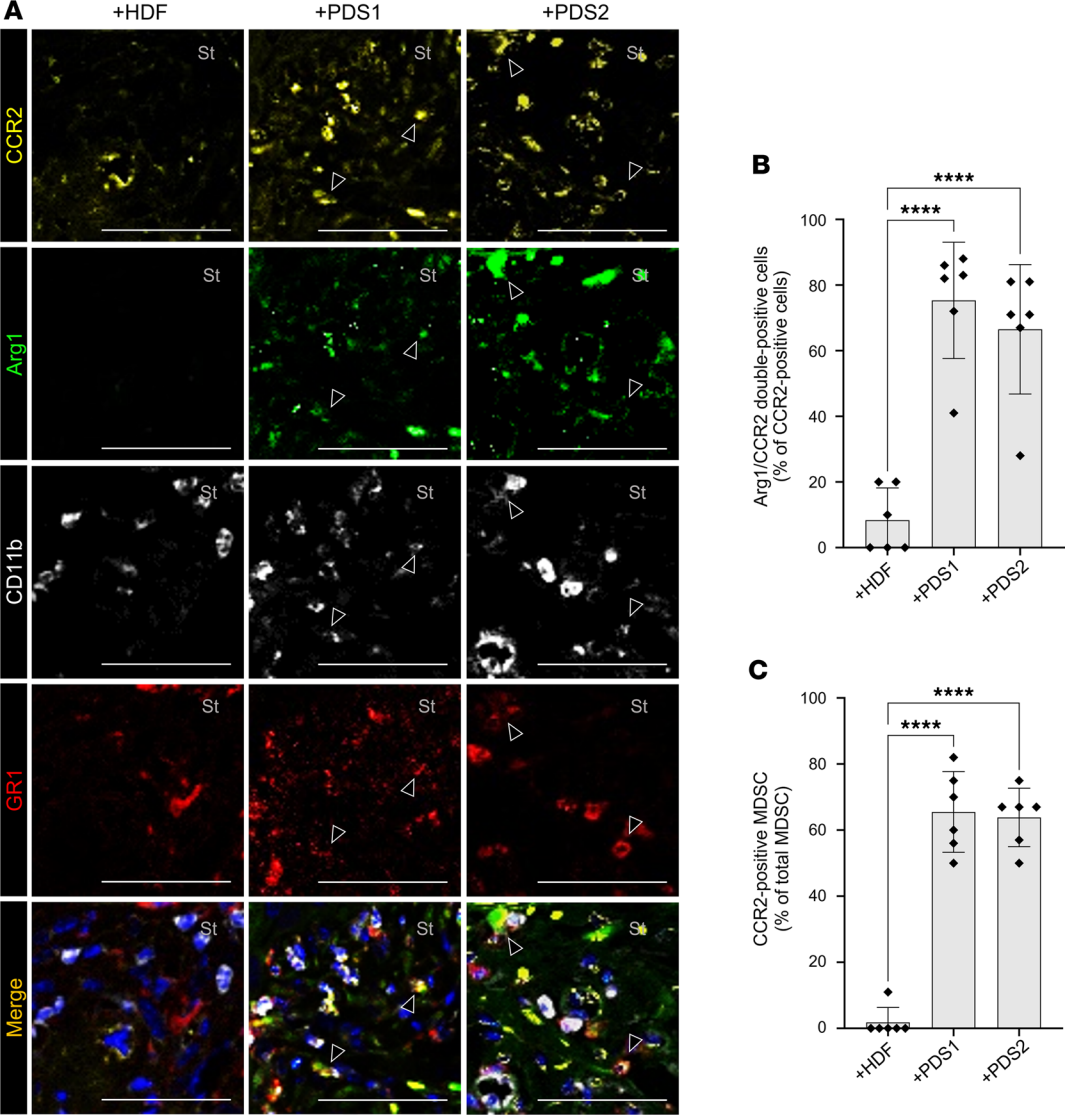
Stroma-derived chemokine CCL2 recruits CCR2^+^ MDSCs into the TME. (**A**) Representative images of multicolor IHC detecting CCR2 (yellow), Arg1 (green), CD11b (white), and GR1 (red). Arrowheads indicate positive stromal cells. Nuclei are stained with DAPI. St, stromal area. Scale bars: 50 μm (**B**) Rate of Arg1^+^CCR2^+^ cells (% of CCR2^+^ cells). (**C**) Rate of CCR2^+^ MDSCs (CCR2^+^Arg1^+^CD11b^+^GR1^+^ cells) per total MDSCs. +HDF, HSC-2 + HDF coxenograft; +PDS1, HSC-2 + PDS1 coxenograft; +PDS2, HSC-2 + PDS2 coxenograft (5 fields per mouse, *n* = 6). All data are shown as mean ± SD. Statistical analyses were performed using 1-way ANOVA followed by Tukey’s multiple-comparison post hoc test; ****P < 0.0001.

**Figure 7:**
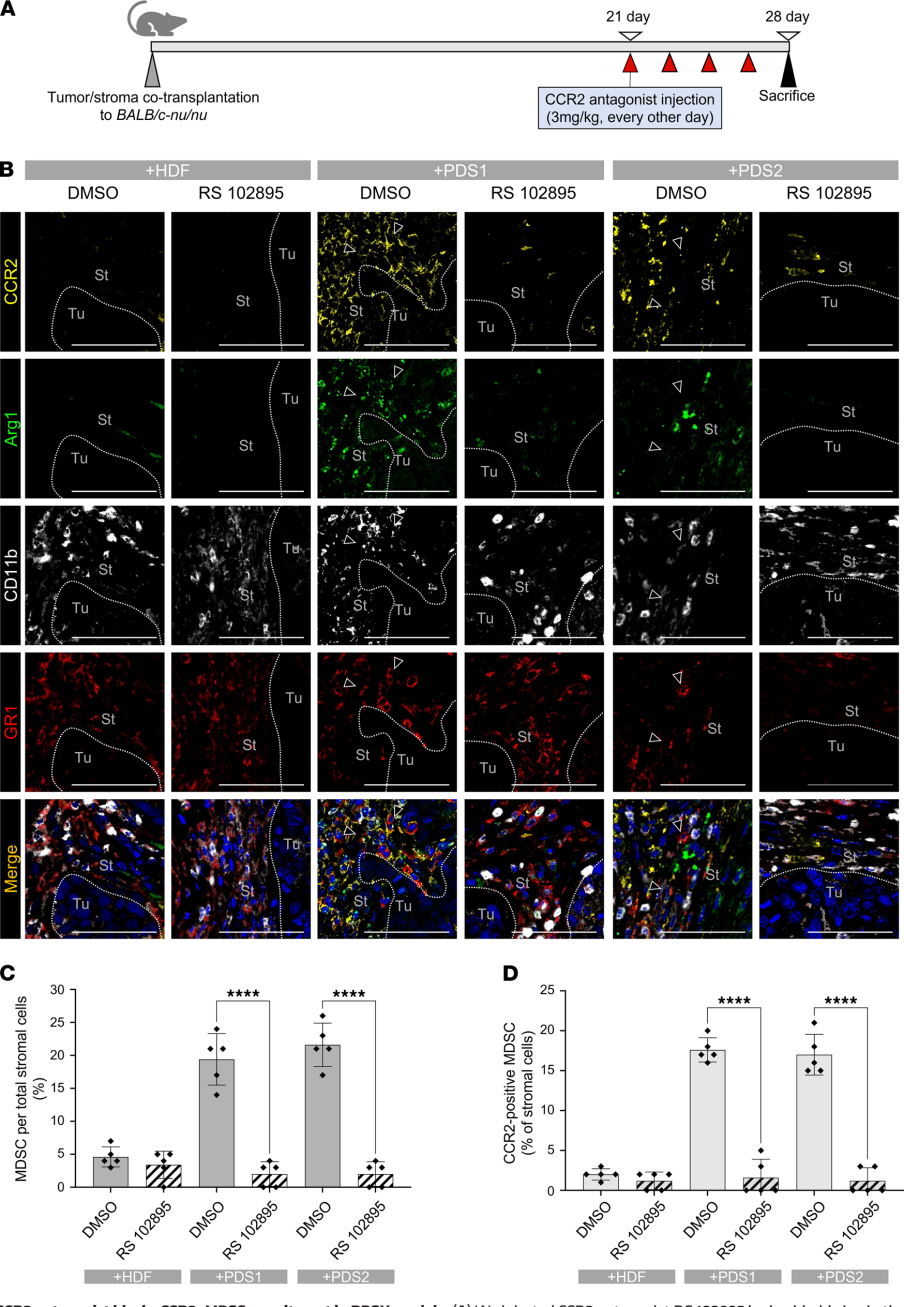
The CCR2 antagonist blocks CCR2^+^ MDSC recruitment in PDSX models. (**A**) We injected CCR2 antagonist RS 102895 hydrochloride i.p. in the concentration of 3 mg/kg (1% DMSO) every other day after 21 days of tumor/stroma cotransplantation. After 1 week, we sacrificed mice and evaluated the CCR2^+^ MDSC recruitment by multicolor IHC staining. (**B**) Representative images of multicolor IHC detecting CCR2 (yellow), Arg1 (green), CD11b (white), and GR1 (red). Arrowheads indicate positive stromal cells. Dotted lines represent the boundary of the tumor (Tu) and stroma (St) area. Nuclei are stained with DAPI. Scale bars: 50 μm. (**C**) Rate of MDSCs (Arg1^+^CD11b^+^GR1^+^ cells) per total stromal cells. (**D**) Rate of CCR2^+^ MDSCs (CCR2^+^Arg1^+^CD11b^+^GR1^+^ cells) per total stromal cells. DMSO, control group injected with DMSO (1%) only. RS 102895, CCR2 antagonist injected group. +HDF, HSC-2 + HDF coxenograft; +PDS1, HSC-2 + PDS1 coxenograft; +PDS2, HSC-2 + PDS2 coxenograft (5 fields per mouse, *n* = 5). All data are shown as mean ± SD. Statistical analyses were performed using Student’s *t* test; ****P < 0.0001.

Next, to determine whether CCR2 was expressed in MDSCs, we performed multicolor immunofluorescence staining for CCR2, Arg1, GR1, and CD11b in the tumor/stromal coxenografts. CCR2^+^ round cells localized to the stromal area of PDSX models, while a few numbers of such cells were found in the HSC-2 + HDF coxenograft (Figure 6A). Notably, among the CCR2^+^ cells localized to the stromal area, the majority coexpressed Arg1 in the PDSX models (75% in HSC-2 + PDS1 and 67% in HSC-2 + PDS2), while only a minority showed such coexpression in the HSC-2 + HDF model (8%) (Figure 6B). Also, cells staining for CD11b and GR1 partially colocalized with those staining for CCR1 in the stroma of PDSX animals. The proportion of CCR2^+^ MDSCs in PDSX animals was significantly higher than the HSC-2 + HDF cotransplanted model (65% in HSC-2 + PDS1, 64% in HSC-2 + PDS2, and 2% in HSC-2 + HDF) (Figure 6C). Furthermore, to investigate whether blocking CCR2 in xenograft models inhibits the recruitment of MDSCs in OSCC TME, we injected CCR2 antagonist i.p. (3 mg/kg, every other day) into the tumor/stromal coxenografts (Figure 7A) and evaluated the MDSCs by multicolor immunofluorescence staining. Interestingly, the CCR2 antagonist significantly reduced the infiltration of CCR2^+^ cells in PDSX groups (Figure 7B). When we quantified our data, the infiltration rate of total MDSCs (Arg1^+^CD11b^+^GR1^+^ cells) significantly decreased in CCR2 antagonist-injected PDSX mice (19%–22%) compared with control PDSX mice (1% DMSO only injection) (2%–3%) (Figure 7C). In addition, the number of CCR2^+^ MDSCs was significantly lower in CCR2 antagonist–injected PDSX animals (1%–2%) compared with control PDSX animals (17%–18%) (Figure 7D). However, CCR2 antagonist injection did not affect MDSCs recruitment; both total MDSCs and CCR2^+^ MDSCs in HSC-2 + HDF cotransplanted models show approximately 2%–5%. These data suggest that CCR2 function is essential for the recruitment of the CCR2^+^ MDSCs to the TME in the PDSX animals and, thus, indicate that the CCL2/CCR2 axis mediates MDSC recruitment to the TME in OSCC.

## Discussion

Tumor cells recruit BMDCs to form a favorable environment for tumor survival and progression (34, 35). In OSCC, the stroma component is abundantly observed and may shape the histological appearance of OSCC (36). However, whether/how resident stroma alters BMDC recruitment in OSCC has not (to our knowledge) been studied. Our PDSX model provides a potentially new approach to reconstructing human carcinoma and TME in living mice. We successfully generated a sequential transplantation model by incorporating (a) GFP-labeled BMT followed by (b) a tumor/stroma coxenograft. In this model, we were able to track the migration of GFP^+^ BMDCs into the TME. Indeed, BMDCs infiltrated the stroma of PDS-derived tumors at a higher rate than that seen with tumor-alone transplantation. Consistent with this observation, coculturing of PDS with cancer cells promoted the migration of BM cells. Thus, using a sequential transplantation model, we demonstrated that stromal cells are essential for the recruitment and migration of BMDCs into the TME.

The present study showed that resident stroma influenced BMDC subsets in the TME, notably by inducing the generation of MDSCs (Supplemental Table 5). Previous works showed that BMDCs could differentiate into CAFs, TECs, TAMs, or MDSCs after reaching the tumor, where the cells then facilitate tumor progression and metastasis (12). However, the potential for BMDCs to differentiate into CAFs (αSMA^+^) and TECs (CD34^+^) is highly variable and depends on tumor type, organ location, and tumor development stage (37, 38). In our model, BMDCs were not able to differentiate into CAFs or TECs, suggesting that the multipotent ability of BMDCs does not relate to the character of the resident stroma. Instead, our data indicate that the resident stroma permits BMDCs to differentiate into TAMs and MDSCs. Murine MDSCs were identified as GR1^+^CD11b^+^ cells and suppress the immune by producing Arg1 (27, 28). While our study highlights the advantages of xenografts of human cancer and stromal cells, it might be pointed out that our current results were obtained in immunocompromised nude mice, which lack thymic glands and in which T cell differentiation is compromised. Although the innate immunity is intact in nude mice, there is a limitation for a detailed study in adaptive immunity (39, 40). We detected the immunosuppressive potential of MDSCs by detection of Arg1 expression. In PDSX, Arg1^+^GR1^+^CD11b^+^ BMDCs highly infiltrated into the TME, suggesting that these cells are MDSCs with immunosuppressive potential. Future studies may need to investigate the relation of cancer-stimulated resident stroma and immunity in detail using another model.

MDSCs are important for immune evasion by tumor cells, allowing the tumor cells to escape immune surveillance. In various types of cancers, inflammatory mediators, including CXC- and CC-motif cytokines, and their functional receptors play important roles in MDSCs recruitment (41). It was reported that, in another tumor xenograft mouse model, tumor types and stages affected the recruitment and the number of MDSCs in the tumor stroma (42). However, previous studies have not investigated whether stromal characters can alter MDSC recruitment. Our cotransplantation model enabled us to determine the role of stromal factors in MDSC recruitment into the TME. Our study determined that the CCL2/CCR2 axis is essential for stroma-driven BM recruitment. The expression of mRNAs encoding several prochemotaxis factors was elevated in the stromal cells, as revealed by microarray analysis. Furthermore, we analyzed whether DEGs of PDS cells from another cancer type were partially common with DEGs of our isolated OSCC-PDSs. From the GEO database, we obtained a gene expression profile of CRC-PDS, a cancer type for which the PDS isolation method (employing enzymatic digestion and ultra-low attachment plates) (31) is similar to our own. OSCC-PDSs and CRC-PDSs shared 9 cellular chemotaxis–related genes out of shared 41 genes. One of the genes, *CCL2*, was upregulated in OSCC PDS1, PDS2, and CRC-PDS. This finding suggested that stroma-derived CCL2 plays a common role in cellular chemotaxis in multiple types of cancers. Moreover, we confirmed the chemotactic role of CCL2 using bindarit, a potent inhibitor of CCL2 production. Indeed, bindarit exposure significantly reduced BM cell migration in the HSC-2 + PDS1/2 coculture (CCL2-high) but not in the HSC-2 + HDF coculture (CCL2-low). Consistently, CCR2 antagonism on BM cells significantly decreased migration in the HSC-2/PDS cocultures. Thus, we concluded that the CCL2/CCR2 axis is essential for the recruitment of CCR2^+^ MDSCs into the TME.

Additionally, it has been reported that the expression of SDF-1 and HGF in cancers supports tumor cell chemotaxis and invasiveness (43). SDF-1 and HGF also have been shown to promote BM stem cell migration to the sites of injury (33, 44). In contrast, our microarray data and in vivo study indicate no significant effect of stromal SDF-1 or HGF in BMDC recruitment to the TME. Together, these data indicate that BM cells are recruited into the oral cancer TME through the action of the stroma factor CCL2 and not by SDF-1 and HGF.

An important finding of our research was that resident stroma–secreted CCL2 directly induces the recruitment of CCR2^+^ MDSCs. Our study models show that the expression of CCL2 in the stroma and its functional receptor, CCR2, on BMDCs were significantly higher in PDSX models compared with the control model. These findings strongly suggest the crucial role of stroma-secreted CCL2 in BM cell migration. Immunosuppressive cells such as MDSCs and TAMs have been shown to express Arg1 (27, 28, 45). Notably, we show that stroma-derived CCL2 recruited CCR2^+^ cells also expressing Arg1, indicating that the migration of immunosuppressive cells into the TME may be mediated by the CCL2/CCR2 axis. Interestingly, multicolor immunostaining revealed that most of CCR2^+^Arg1^+^ cells were MDSCs, and recruitment of CCR2^+^ MDSCs was increased in CCL2-high PDSX models. Moreover, injection of the CCR2 antagonist to mice showed a significant decrease in the infiltration of total MDSCs and CCR2^+^ MDSCs in CCL2-high PDSX models, suggesting that the CCL2/CCR2 axis governs recruitment of MDSCs into OSCC TME. Consistent with our observation, many studies have reported a role for the cancer-secreted CCL2 in recruiting CCR2^+^ MDSCs to the TME (16). Nevertheless, using the original cotransplantation model, we reported here for the first time to our knowledge that resident stromal cells that secrete CCL2 are essential for the recruitment of MDSCs.

Taken together, our study suggests that the targeting of a key stromal factor likely will serve as a crucial strategy for the treatment of OSCC. The interaction of stroma-secreted CCL2 with CCR2^+^ MDSCs is essential for the complex crosstalk between cancer cells, stromal cells, and MDSCs; thus, it promises to constitute an attractive target for OSCC treatment.

## Methods

### Cell line and mice.

Human OSCC cell line (HSC-2; JCRB0622) and HDF (normal HDF; adult [NHDF-Ad; CC-2511]; batch no. 0000477954) were purchased from JRCB Cell Bank and Lonza Walkersville, respectively. Cells were grown in minimum essential medium-α (α-MEM) (Invitrogen) with 10% FBS (Invitrogen) and 1% antibiotic/antimycotic (Invitrogen) at 37°C in a humidified atmosphere with 5% CO_2_. Female nude mice (*BALB/c-nu/nu* and *C57BL/6-BALB/c-nu/nu-GFP* Tg mice) purchased from Shimizu Laboratory Suppliers were housed under pathogen-free conditions.

### PDS cells.

For PDS cell isolation, the primary culture of human OSCC tissues, and subsequent isolation of the stromal cells were performed according to the protocol described previously (36). Firstly, we obtained the fresh tissue specimens (1 mm^3^) from 2 patients suffering from OSCC in the tongue. After washing with α-MEM (Invitrogen) containing 1% antibiotic/antimycotic (Invitrogen) several times, the tissue specimens were minced into pieces. Then, the enzymatic digestion of tissue specimens was performed by treating with α-MEM (Invitrogen) containing collagenase II (1 mg/mL) (Invitrogen) and Dispase (1 mg/mL) (Invitrogen) for 2 hours at 37°C with shaking (200 rpm). The released cells were centrifuged at 111.8*g* for 5 minutes at room temperature (RT), suspended in α-MEM (Invitrogen) containing 10% FBS (Invitrogen), filtered using a cell strainer (100 μm, BD Falcon), plated in a tissue culture flask, and incubated at 37°C in a humidified atmosphere with 5% CO_2_. After 1 week, the primary stromal cells mixed with cancer cells were separated by treating with Accutase (Invitrogen) based on the different adherence ability between epithelial and stromal cells. We named isolated stromal cells as PDS cells (PDS1 and PDS2). Cells were maintained and exposed to primary culture in α-MEM (Invitrogen) with 10% FBS (Invitrogen) and 1% antibiotic/antimycotic (Invitrogen) at 37°C in a humidified atmosphere with 5% CO_2_. We obtained informed consent from all patients.

### Isolation of BM cells.

For in vitro experiments, we performed a primary culture of BM cells. We collected the femur and tibia from 8-week-old GFP transgenic nude mice and removed the soft tissues from the bone. Then, proximal parts of bone tissues were cut. The BM space was washed out with α-MEM (Invitrogen) until the bone color turned white. The obtained BM cells were plated in α-MEM (Invitrogen) with 10% FBS (Invitrogen) and 1% antibiotic/antimycotic (Invitrogen) at 37°C in a humidified atmosphere with 5% CO_2_.

### Wound closure assay.

We prepared different conditioned media from cocultures of HSC-2 and stromal cells (HDF, PDS1, or PDS2) or HSC-2 cells alone. To prepare the conditioned media, tumor cells and stromal cells were plated in a ratio of 1:3 and cultured for 24 hours. The media were replaced with fresh serum-free α-MEM (Invitrogen). Cells were further cultured for 48 hours, and then conditioned media were collected and filtered with a 0.2 μm–pore filter (Steradisc 25, Kurabo). For the migration assay, isolated BM cells were seeded in a 6-well plate and grown for 24 hours to approximately 90% confluence. The medium was removed, and cell monolayers were wounded by manual scraping with a sterile P200 micropipette tip. Debris was washed off with PBS 3 times. Cells were then cultured in different conditioned media with 10% FBS (Invitrogen) at 37°C in a humidified atmosphere with 5% CO_2_. Images were captured immediately and subsequently at 3, 6, and 9 hours using an Eclipse Ts2-FL microscope (Nikon). The percentage of the area closed was calculated using ImageJ (NIH, v1.52a). Independent experiments were repeated 3 times.

### Transwell migration assay.

HSC-2 (6 × 10^4^) with or without stromal cells (24 × 10^4^) that were suspended in α-MEM (750 μL) containing 10% FBS were seeded in 24-well plates for 24 hours. BM cells (0.5 × 10^6^) suspended in serum-free α-MEM (300 μL) were placed into the upper inserts with a pore size of 8 μm. Following incubation for 9 hours at 37°C, Giemsa staining was performed using Diff-Quik solution (Nanjing Jiancheng Bioengineering Institute), and nonmigrating cells were generally removed with cotton swabs. Photomicrographs of migrating cells were taken using an upright microscope BX53 (Olympus). Independent experiments were repeated 3 times, and data were quantified with ImageJ (v1.52a).

Transwell migration was analyzed using CCL2 synthesis inhibitor, bindarit (Cayman Chemical Company), or CCR2 antagonist RS 102895 hydrochloride (Cayman Chemical Company). Bindarit was prepared in dimethyl sulfoxide (DMSO), as described previously (46). Briefly, a 50 mM stock solution of bindarit was ready in DMSO and diluted to 50 μM (0.1% DMSO), 100 μM (0.2% DMSO), and 300 μM (0.6% DMSO) in the culture medium of the lower chamber where HSC-2 + HDF, HSC-2 + PDS1, and HSC-2 + PDS2 were seeded. As a control, cells were seeded in 0.6% DMSO. After incubation with the inhibitor for 4 hours at 37°C, the migration of BM cells was allowed for 9 hours at 37°C. For CCR2 antagonism, BM cells were pretreated with RS 102895 (20 μM) prepared in DMSO (0.04%) for 1 hour and then suspended in α-MEM (300 μL). The cells were then seeded into the upper chamber and allowed to migrate to the lower chamber of tumor/stroma cocultures for 9 hours at 37°C. Giemsa staining was performed. Migrating cells were quantified as described above. Independent experiments were repeated 3 times.

### BMT.

To trace the BMDCs in the TME, we performed BMT to nude mice with GFP^+^ total BM cells, according to a standard protocol as described previously (34). As described above, BM cells were freshly collected from GFP nude mice and suspended within HBSS (Invitrogen) at a concentration of approximately 1.0 × 10^7^ cells/0.2 mL. Meanwhile, 8-week-old female nude recipient mice were subjected to 8 Gy of lethal whole-body irradiation (4 Gy, 2 times), and BM cells were injected into the tail vein.

### Xenograft and PDSX mice.

Tumor transplantation was subjected to BMT nude mice after 4 weeks of BMT as described previously (35). Four types of tumor models (*n* = 6 for each) were created; HSC-2–only transplantation (HSC-2), HSC-2 and HDF transplantation (HSC-2 + HDF), and HSC-2 and PDS transplantation (HSC-2 + PDS1 and HSC-2 + PDS2). Monolayers of tumor cells and stromal cells were detached by trypsinization, washed, and suspended in HBSS (Invitrogen) at the concentrations of 1.0 × 10^6^ HSC-2 cells per 0.2 mL and 3.0 × 10^6^ stromal cells per 0.2 mL. For the tumor-alone model, 1.0 × 10^6^ HSC-2 cells were injected s.c. to the head. For HSC-2 + HDF, HSC-2 + PDS1, and HSC-2 + PDS2 models, the mixtures of tumor cells and stromal cells were prepared at a ratio of 1:3 (1.0 × 10^6^ HSC-2 cells and 3.0 × 10^6^ stromal cells) and injected s.c. to the head. At 28 days, all mice were euthanized, and the specimens were harvested for analysis.

### Administration of CCR2 to mice.

For blocking CCR2, after 21 days of tumor transplantation of HSC-2 + HDF, HSC-2 + PDS1, and HSC-2 + PDS2 (*n* = 5 for each), i.p. injection of CCR2 antagonist (RS 102895) dissolved in DMSO at a final concentration of 3.0 mg/kg (1% DMSO) was given to mice for 1 week (every other day). As a control, i.p. injection of 1% DMSO was performed. After 1 week, mice were euthanized, and the specimens were harvested for analysis.

### Tissue processing for histological examination.

For the preparation of formalin-fixed paraffin-embedded sections, the harvested tumor tissues and bone were fixed in 4% paraformaldehyde for 12 hours, and decalcification of bone was performed in 10% EDTA at 4°C for 14 days. Then, samples were consequentially dehydrated in 70% ethanol and embedded in paraffin. Serial sections (3 μm) were prepared. Sections were stained with H&E, IHC, and fluorescent IHC.

### IHC.

IHC was carried out using the antibodies detailed in Supplemental Table 6. After the antigen retrieval, sections were treated with 10% normal serum for 15 minutes and then incubated with primary antibodies at 4°C overnight. Signals were enhanced by the avidin-biotin complex method (Vector Lab). Color development was performed with DAB (Histofine DAB substrate), and the staining results were observed with an optical microscope (BX53, Olympus).

### Double-fluorescent IHC.

Following the antigen retrieval, sections were incubated in Block Ace (DS Pharma Biomedical) for 20 minutes at RT, followed by incubation with primary antibodies at 4°C overnight. The secondary antibody application was performed at a dilution of 1:100 for 1 hour at RT. The secondary antibodies used are detailed in Supplemental Table 7. The sections were then stained with 0.2 g/mL of DAPI (Dojindo Laboratories). The staining results were observed with an All-in-One BZ x700 fluorescence microscope (Keyence).

### Multicolor fluorescent IHC.

For MDSCs detection, the OPAL 7-color manual IHC kit was used according to the manufacturer’s instructions (PerkinElmer). Briefly, antigens were detected separately by performing the repeated cycles of the staining procedure. After antigen retrieval, sections were incubated in a blocking agent for 10 minutes and were incubated with primary antibodies at 4°C overnight or 37°C for 2 hours. Then, sections were treated with secondary-HRP for 10 minutes and signal amplification with Opal-fluorophore for 10 minutes. Antigen stripping was performed by microwave heating before the next target detection, and the procedure was repeated for the next target from the blocking step. After detecting all the targets, sections were counterstained with DAPI, and sections were visualized using a ZEISS LSM 700 confocal microscope (Germany).

### Gene expression analysis.

HDF and PDS1/2 cells were cultured for 3 days, and total RNA was isolated from cultured cells using RNeasy Mini Kit (QIAGEN). Concentration and quality (A260/280) of total RNA were measured by Bioanalyzer (Agilent, 2100). cDNA was synthesized from 0.1 μg of total RNA using a Low Input Quick Amp Labelling Kit (Agilent Technologies) and was then hybridized to probes of Agilent Technologies Microarray (SurePrint G3 Human 8x60K Ver.3.0). Microarray data are available in NCBI GEO (GSE164374). Gene expression of HDF versus PDS1/2 was compared, and DEGs with more than 3 SDs were selected for further analysis. As for the validation data set, we additionally analyzed the publicly available independent data set (GSE93253) and compared expression profiles of NF (*n* = 4) versus CRC-PDS (*n* = 3). The same cut-off (3 SDs) was applied for comparison between NF and CRC-PDS. Venn diagrams were generated using the Venny 2.1. Genes related to cellular chemotaxis were selected using DAVID Bioinformatics Resources 6.8. Bubble plot presentation of biological process enrichment analysis was performed by R (3.6.2). Bar chart and heatmap presentation of DEGs of HDF versus PDS1/2 was generated using Microsoft Excel.

### Whole cell lysate.

Cells were cultured until being subconfluent and then washed with PBS (–), treated with 100 to 200 μL/10 cm dish of a 1× RIPA buffer (Cell Signaling Technology) in PBS (–) supplemented with a protease inhibitor cocktail and PMSF, and collected by using a cell scraper. Cells were further lysed by a 25 G needle syringe for 10 strokes and then incubated overnight with shaking at 4°C. The lysate was centrifuged at 10,000*g* for 20 minutes at 4°C, and the supernatant was used as a whole-cell lysate (WCL). The WCL was diluted 10-fold, and protein concentration was measured using the BCA protein assay system (Thermo Fisher Scientific).

### Western blotting.

Western blotting was performed as described previously (47). A total of 5 μg protein was mixed with 4× Laemmli sample buffer (Bio-Rad Laboratories) and boiled at 95°C for 5 minutes. Each protein sample was separated by SDS-PAGE in 4%–20% TGX-GEL (Bio-Rad Laboratories) and transferred to polyvinylidene difluoride (PVDF) membranes (Bio-Rad Laboratories) by using a semidry method. The membranes were blocked in 5% skim milk in Tris-buffered saline (TBS) containing 0.05% Tween 20 (TBS-T) for 1 hour with shaking at RT. Afterward, the membrane was incubated overnight with shaking at 4°C with primary antibodies, which cross both human and mice — rabbit anti–E-cadherin (1:1000, 24E10, Cell Signaling Technology), rabbit anti-vimentin (1:1000, Abcam), or rabbit anti-FAP (1:500, Abcam) — and then incubated with horseradish peroxidase–conjugated (HRP-conjugated) secondary antibodies for 1 hour with shaking at RT. Washes before and after antibody reactions were done on a shaker, 3 times within TBS-T for 10 minutes each at RT. Alternatively, before the detection of β-actin, we performed the stripping procedure for 10 minutes because the molecular weight of vimentin and β-actin are similar. Then, the membranes were incubated with HRP-conjugated mouse anti–β-actin (1:20,000, Abcam) antibody for 1 hour with shaking at RT. Blots were visualized with ECL substrate (Bio-Rad Laboratories).

### Statistics.

In tumor tissues, we performed quantification and found the average on 5 randomly captured images of stromal area at the magnification of 400× per mouse (*n* = 6 for each OSCC model, *n* = 5 for each CCR2 antagonist injection or DMSO-only injection model). For quantification of transwell migration, we performed independent experiments 3 times (*n* = 3), and quantification was done on 5 randomly captured images per group at each time. For the wound healing assay, we repeated the independent experiment 3 times (*n* = 3), and the average migrating area was calculated on 5 images per group at each time. The counting and area measurement was performed using ImageJ (v1.52a).

All statistical analyses were conducted using GraphPad Prism 9.1.1. In short, 2-tailed Student’s *t* test for independent samples with equal variances was used to compare 2 groups, while 1-way ANOVA followed by Tukey’s multiple-comparison post hoc test was used to compare differences between more than 2 groups where necessary. Differences were considered significant at *P* < 0.05. Data were presented as mean ± SD.

### Study approval.

All animal experiments were undertaken by the guidelines of the Okayama University Care and Use of Laboratory Animals. This research was approved by the Committee on the Ethics of Animal Experiments of Okayama University Graduate School of Medicine, Dentistry and Pharmaceutical Sciences (OKU-2017406). PDS cells isolation was approved by the Ethics Committee of Okayama University (approval no. 1703-042).

## Author contributions

MWO performed BM and tumor transplantation, performed primary cell culture, performed IHC, performed microarray analysis, performed data analysis, and wrote the original draft of the manuscript. HK conceptualized, designed, and managed the study; performed BM and tumor transplantation, IHC, primary cell culture, and data analysis; wrote the original draft of the manuscript; and acquired funding. KT performed BM and tumor transplantation, performed primary cell culture and data analysis, and acquired funding. ST performed microarray analysis and edited the manuscript. TE, SS, and TO wrote and edited the manuscript. K Ono performed Western blotting. QS performed microarray analysis. SY and HO performed IHC and data analysis and acquired funding. KN edited the manuscript and acquired funding. HN, K Okamoto, and AS supervised HK and MWO. All authors have read and agreed to the published version of the manuscript.

## Supplementary Material

Supplemental data

## Version 1 12/07/2021

In-Press Preview

## Version 2 01/11/2022

Electronic publication
