# Supplementary material for: Resident stroma-secreted chemokine CCL2 governs myeloid-derived suppressor cells in the tumor microenvironment
Source: JCI Insight. 2022 Jan 11;7(1):e148960. doi: 10.1172/jci.insight.148960 (PMC8765046; doi:10.1172/jci.insight.148960)
Supplement: Supplemental data [file jciinsight-7-148960-s130.pdf]

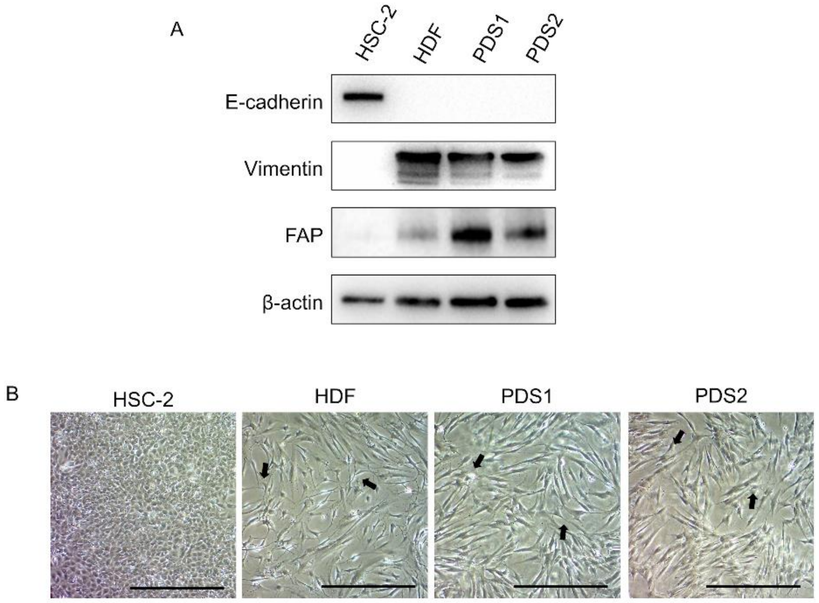

Supplementary Figure 1. Isolation of PDS from oral squamous cell carcinoma tissue specimens: (A) Western blot showing E-cadherin, vimentin, and fibroblast activated protein (FAP) expression in HSC-2, HDF, PDS1, and PDS2. HSC-2 and HDF were used for positive control of E-cadherin and vimentin, respectively. Whole-cell lysate and culture supernatant were prepared from the cells cultured in a serum-free medium for 24 h. 20 µg protein sample was applied per lane. All antigen-antibody reactions were performed using the same membrane. There was no contamination of tumor cells (no expression of E-cadherin) in the isolated PDSs, showing the expression of vimentin and FAP. **(B)** Representative images of HSC-2, HDF, isolated PDS1, and PDS2. Arrows indicate the intercellular connection of cells. Scale bars, 500 µm.

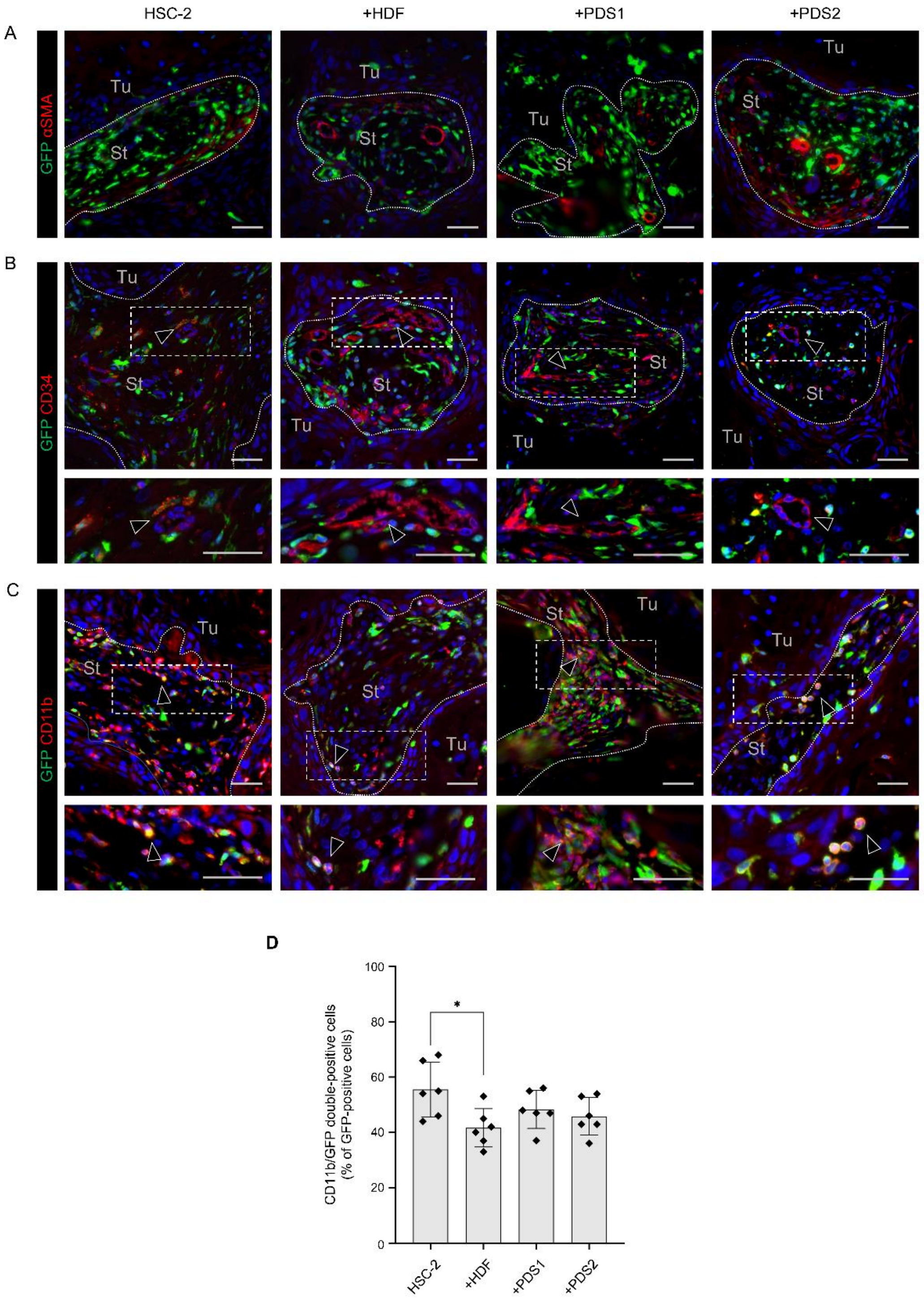

Supplementary Figure 2. The resident stroma influences the potential of BMDCs differentiation into TAMs but not on CAFs and TECs in OSCC. The tumor tissue sections of HSC-2, +HDF, +PDS1, and +PDS2 were analyzed for αSMA-positive CAFs, CD34-positive TECs, and CD11b-positive TAMs co-staining with GFP-positive BMDCs. **(A)** Representative images of double-fluorescent IHC for GFP (green) and αSMA (red). **(B)** Representative images of double-fluorescent IHC for GFP (green) and CD34 (red). Images on the bottom, high-magnified images. Arrowheads indicate CD34-positive vessel loop formation. **(C)** Representative images of double-fluorescent IHC for GFP (green) and CD11b (red). Insets are high-magnified images. Arrowheads indicate CD11b/GFP double-positive cells. **(D)** The rate of CD11b/GFP double-positive cells (% of GFP-positive cells). Dotted lines represent the boundary of the tumor (Tu) and stroma (St) area. Nuclei are stained with DAPI. Scale bars, 50 μm. HSC-2, HSC-2 alone xenograft. +HDF, HSC-2+HDF co-xenograft. +PDS1, HSC-2+PDS1 co-xenograft. +PDS2, HSC-2+PDS2 co-xenograft (5 fields per mouse, n = 6). All data are shown as Mean ± SD. Statistical analyses were performed using one-way ANOVA followed by Tukey's multiple comparison post hoc test; * $P < 0.05$.

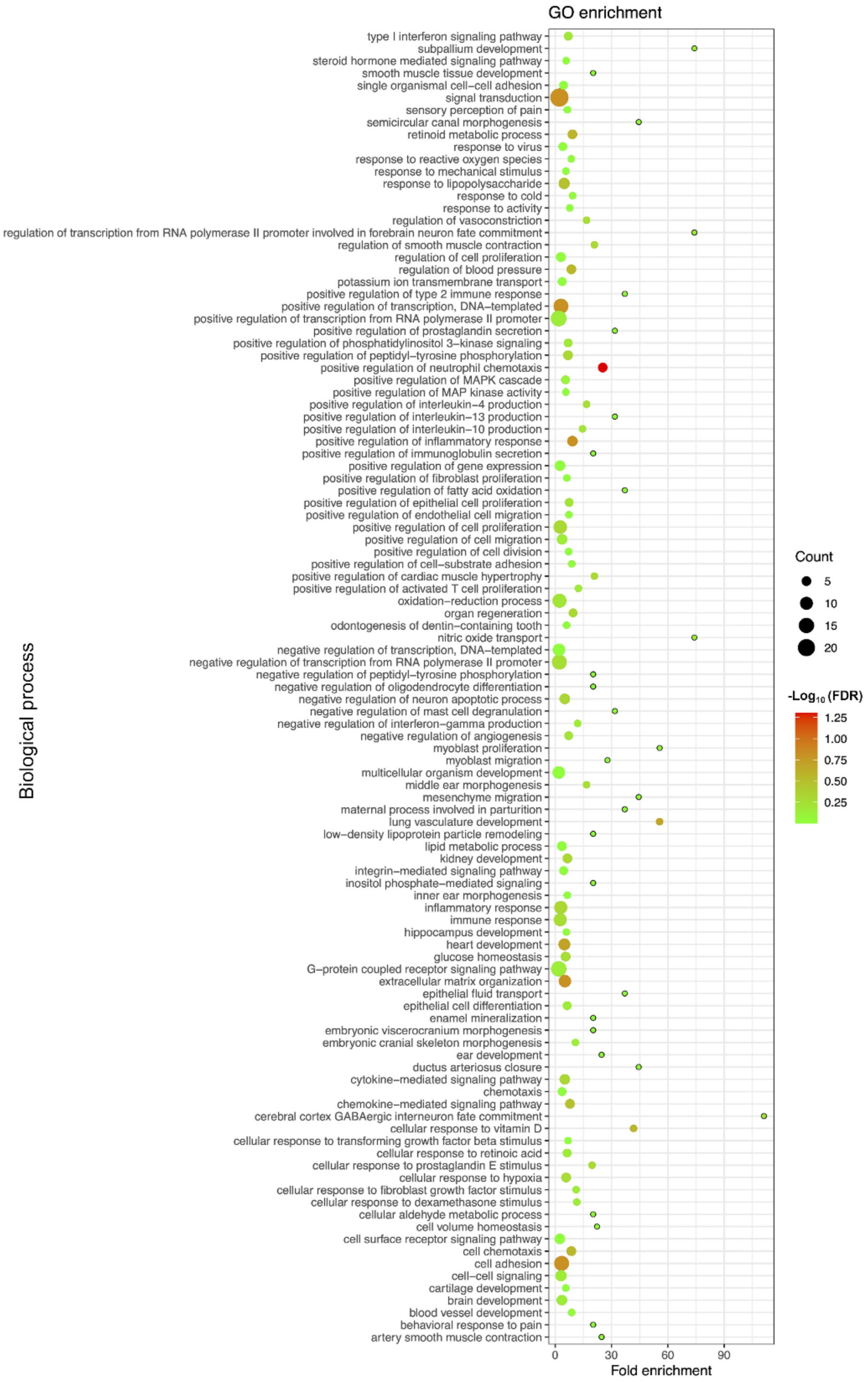

Supplementary Figure 3. The biological process enrichment analysis of common 193 differentially expressed genes (DEGs) of oral cancer PDS1 and PDS2. The biological process enrichment analysis was performed using DAVID and presented with a bubble plot. The color of each bubble represented the false discovery rate (FDR), and the size of the bubble represented the count of the genes.

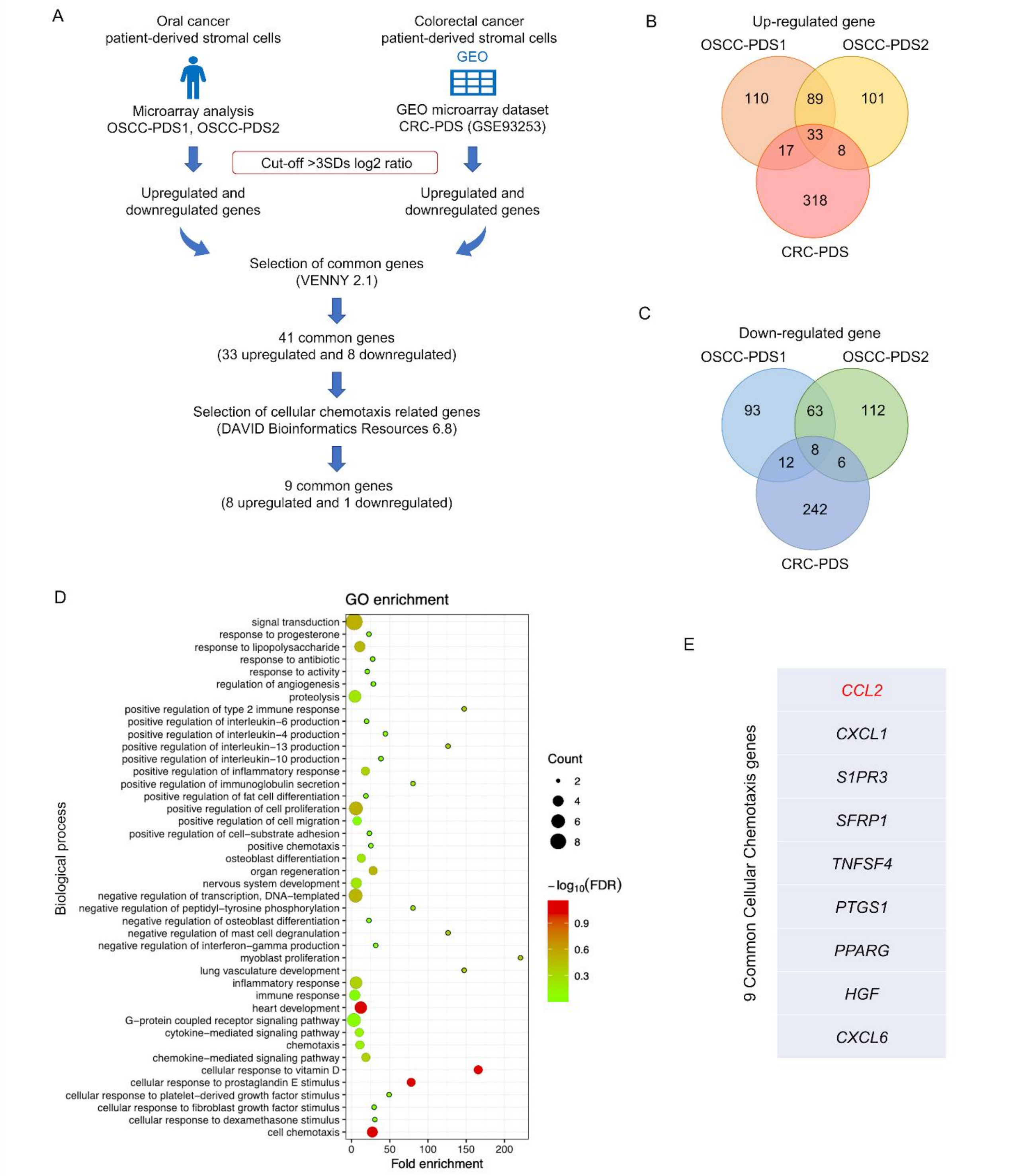

Supplementary Figure 4. Gene expression profiles of OSCC-PDSs and CRC-PDS shared 9 cellular chemotaxis-related genes. **(A)** Flow chart of cellular chemotaxis-related genes screening. We analyzed the differentially expressed genes (DEGs) of oral squamous cell carcinoma (OSCC) PDS1 and PDS2 compared to HDF (upper left). We screened the GEO database and analyzed the DEGs of other cancer. We selected colorectal cancer (CRC) PDS from GEO (GSE93253), which has the same stroma isolation method. (Upper right). Then we compared DEGs of OSCC-PDSs and CRC-PDSs and selected the common DEGs by Venny 2.1. Then, we observed the cellular chemotaxis-related genes by DAVID bioinformatics Resources 6.8. **(B)** Venn diagram for commonly upregulated gene expression among OSCC-PDS1, OSCC-PDS2, and CRC-PDS. **(C)** Venn diagram for commonly downregulated gene expression among OSCC-PDS1, OSCC-PDS2, and CRC-PDS. **(D)** The biological process enrichment analysis of common 41 DEGs was performed using DAVID and presented with a bubble plot. The color of each bubble represented the false discovery rate (FDR), and the size of the bubble represented the count of the genes. **(E)** Table of 9 common cellular chemotaxis-related genes. *CCL2* is included in the shared DEG of OSCC-PDS and CRC-PDS.

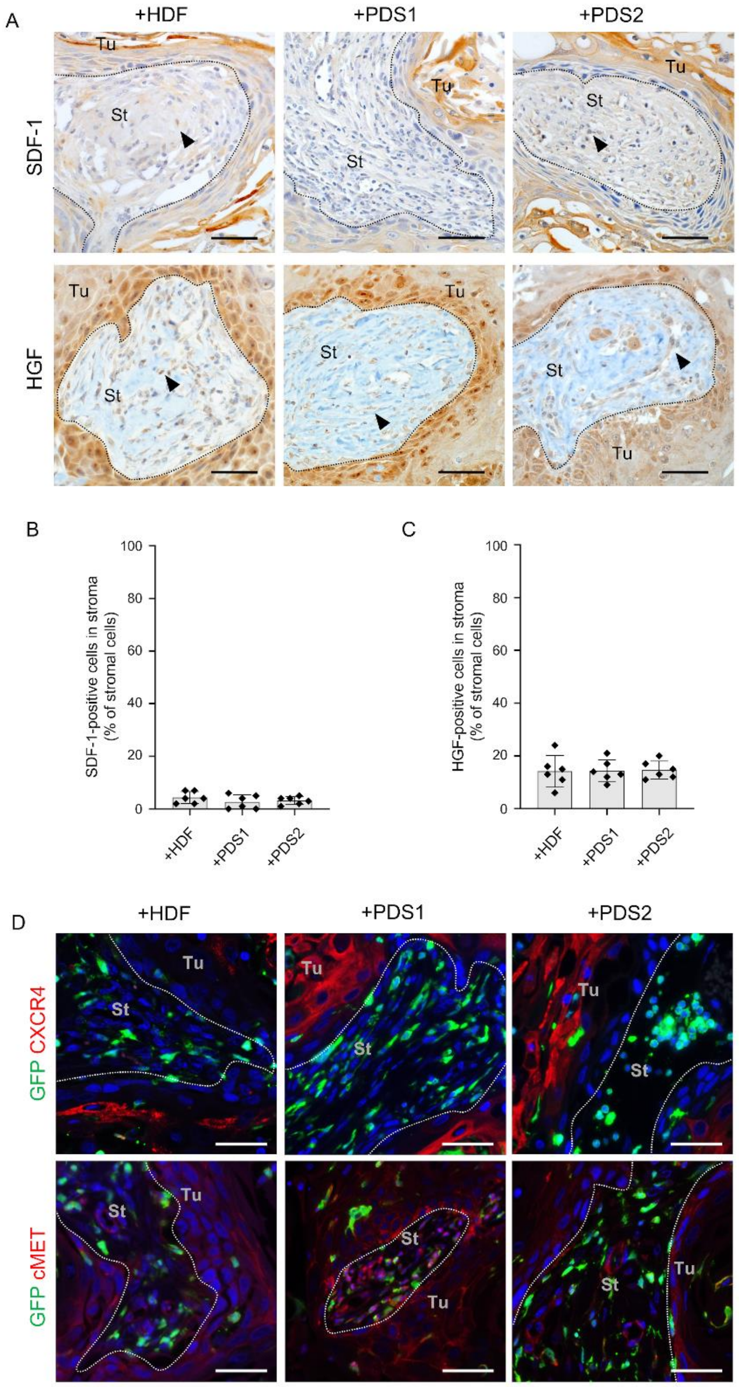

Supplementary Figure 5. SDF1 and HGF are the weak stromal secreted factors for BMDCs recruitment in OSCC. (A) IHC features of SDF-1 (upper panels) and HGF (lower panel). Arrows indicate positive stromal cells. Dotted lines represent the boundary of the tumor (Tu) and stroma (St) area. Scale bars, 50 μm. **(B)** The rate of SDF-1-positive cells in the stroma. **(C)** The rate of HGF-positive cells in the stroma. The positive cells were calculated as the percentage of total stromal cells using Image J software. **(D)** Representative images of double-fluorescent IHC for GFP (green) and CXCR4 (red) (upper panels) or cMet (red) (lower panels). Dotted lines represent the boundary of the tumor (Tu) and stroma (St) area. Nuclei are stained with DAPI. Scale bars, 50 μm. +HDF, HSC-2+HDF co-xenograft. +PDS1, HSC-2+PDS1 co-xenograft. +PDS2, HSC-2+PDS2 co-xenograft (5 fields per mouse, n = 6). All data are shown as Mean ± SD. Statistical analyses were performed using one-way ANOVA followed by Tukey's multiple comparison post hoc test.

Up-regulated genes with more than 3SD in PDS1											
1	A_33_P3212316	46	CXCL3	91	HGF	136	lnc-STIL-1	181	PRRG1	226	THBS4
2	ABCB4	47	CXCL6	92	HLA-B	137	lnc-TMEM135-2	182	PSG8	227	TMEM176A
3	ACTG2	48	CYP1B1	93	HSD11B1	138	lnc-TMEM85-1	183	PTGS1	228	TMEM30B
4	ADAMTS8	49	DCN	94	HSD17B6	139	lnc-UMODL1-1	184	PTN	229	TMTC1
5	ADCY4	50	DDX58	95	ICAM5	140	LOC100128340	185	RAET1E-AS1	230	TNFSF13B
6	ADRB2	51	DENND2A	96	ID1	141	LOC100288798	186	RAMP1	231	TNXB
7	AKR1B10	52	DKFZp779M0652	97	IFI27	142	LOC101927668	187	RASGRP3	232	TP53I11
8	ALDH1A1	53	DLX1	98	IFI44L	143	LRRN2	188	RASL11A	233	TSPAN11
9	ANKRD29	54	DLX2	99	IFI6	144	LYPD1	189	RASL11B	234	TUBA4A
10	ANO3	55	DOK6	100	IFIH1	145	MAOA	190	RDH10	235	TUSC1
11	APBB1IP	56	EDN1	101	IFIT2	146	MECOM	191	RGCC	236	UBL4B
12	APOD	57	EEPD1	102	IFIT3	147	MFSD6	192	RGS2	237	USP18
13	ARHGAP20	58	EGFL6	103	IGF2	148	MGAT3	193	RGS9	238	VIT
14	ATF7IP2	59	ENST00000414768	104	IGFBP2	149	MGAT5	194	RSAD2	239	WDR5
15	ATOH8	60	ENST00000438824	105	IGFBP5	150	MITF	195	S1PR3	240	WLS
16	ATP8B4	61	ENST00000440492	106	IL13RA2	151	MMP12	196	SAMD5	241	WNT11
17	BEX1	62	ENST00000479351	107	IL32	152	MN1	197	SCAMP1-AS1	242	WNT16
18	BIRC7	63	ENST00000487097	108	IL33	153	MX1	198	SCN4B	243	WWC1
19	BMP2	64	ENST00000522356	109	ISOC1	154	MX2	199	SCN9A	244	XAF1
20	BMP6	65	ENST00000554954	110	ITGA8	155	NCKAP5	200	SEMA3A	245	XLOC_l2_011873
21	BST2	66	ENST00000567054	111	ITGB2	156	NOVA1	201	SEPP1	246	XLOC_l2_014549
22	C15orf48	67	ENST00000590085	112	ITPR1	157	NR2F1	202	SERINC2	247	ZDHHC11
23	C9orf47	68	EPB41L4B	113	JRKL-AS1	158	NR4A3	203	SESN3	248	ZDHHC8
24	CABLES1	69	EPSTI1	114	KBTBD11	159	OAS1	204	SFRP1	249	ZNF577
25	CACNA1G	70	ETV1	115	KCNJ2	160	OAS2	205	SH3RF3-AS1		
26	CAMK1D	71	EXOC6	116	KCNJ8	161	OAS3	206	SHC3		
27	CCL2	72	F2R	117	KIAA1644	162	OASL	207	SHC4		
28	CCL5	73	FAM107B	118	KRT32	163	OLFM1	208	SIX1		
29	CCR7	74	FAM131B	119	LAMA3	164	PCSK5	209	SIX2		
30	CD36	75	FAM43A	120	LCE2A	165	PDK4	210	SLC12A7		
31	CD9	76	FAM65C	121	LCE2C	166	PENK	211	SLC16A6		
32	CDH6	77	FBLN1	122	LCE3B	167	PGM5	212	SLC2A12		
33	CECR1	78	FBXO15	123	LINC00441	168	PHLDA1	213	SLC40A1		
34	CES1	79	FOXF1	124	LINC00578	169	PITPNC1	214	SLC43A2		
35	CHI3L1	80	FOXF2	125	LINC00671	170	PITX1	215	SMOC1		
36	CHRDL1	81	GALNT18	126	LINC00842	171	PLA2G2F	216	SNAP25		
37	CHRM2	82	GCA	127	LINC01111	172	PLA2G4A	217	SOD3		
38	CHURC1	83	GMPR	128	LINC01291	173	PLAU	218	SPAG16		
39	CLGN	84	GPC4	129	LINC01436	174	PNMA2	219	STAMBPL1		
40	CMPK2	85	GPRC5B	130	lnc-AP000769.1-1	175	PNPLA3	220	STRA6		
41	CNOT4	86	GPX3	131	lnc-CLVS1-1	176	PPAP2C	221	SVEP1		
42	COLEC11	87	GREM2	132	lnc-FZD4-1	177	PPARG	222	SYBU		
43	CPED1	88	GRIA3	133	lnc-GMDS-2	178	PPP4R4	223	TFPI2		
44	CXCL1	89	HAND2-AS1	134	lnc-OR1Q1-1	179	PRDM16	224	TGFB2		
45	CXCL2	90	HECW2	135	lnc-SLC4A3-3	180	PRKCH	225	TGM2		

Supplementary Table 1. Upregulated genes with more than 3SDs in PDS1. Gene expression of PDS1 was analyzed by the Agilent microarray method. Comparing with HDF, fold-change, 3SDs Log2(fold-change) were calculated.

Up-regulated genes with more than 3SD in PDS2											
1	A_33_P3240078	46	CXCL2	91	GSTT2B	136	LOC101930611	181	S1PR3	226	WLS
2	A_33_P3245126	47	CXCL3	92	GUSBP1	137	LOC102724074	182	SAA1	227	WNT16
3	ABHD11-AS1	48	CXCL6	93	GYG2	138	LOC102724348	183	SAMD5	228	WWC1
4	ACTG2	49	CXCL8	94	HAND2-AS1	139	LOC441268	184	SCN9A	229	XLOC_l2_011873
5	ADAMTS9	50	DAPK2	95	HCLS1	140	LRRD1	185	SERINC2	230	XPNPEP2
6	ADRB2	51	DDX60L	96	HECW2	141	LRRN2	186	SERPINB9	231	ZNF503-AS2
7	AFF3	52	DIO2	97	HGF	142	LTBP1	187	SESN3		
8	AK024445	53	DKFZp779M0652	98	HLA-B	143	MAOA	188	SFN		
9	AKAP12	54	DLX1	99	HLA-DPA1	144	MASP1	189	SFRP1		
10	AKR1B10	55	DLX2	100	HLA-DPB1	145	MECOM	190	SHC3		
11	ALDH1A1	56	DOK5	101	ICAM1	146	MFSD6	191	SHC4		
12	ANKRD29	57	EDN1	102	ICAM5	147	MITF	192	SIX1		
13	ANO3	58	EGFL6	103	ID1	148	MMP1	193	SLC12A7		
14	APBB1IP	59	EIF4G3	104	IFI27	149	MN1	194	SLC16A6		
15	APOD	60	ELOVL5	105	IGF2	150	MX1	195	SLC25A27		
16	AR	61	EML1	106	IL13RA2	151	NR2F1	196	SLC2A12		
17	ARHGAP20	62	ENST00000423466	107	IL32	152	NR4A3	197	SLC39A8		
18	ARHGAP5-AS1	63	ENST00000440528	108	IL33	153	OAS1	198	SLC40A1		
19	ARHGDIB	64	ENST00000453852	109	IL6	154	OLFM1	199	SLC43A2		
20	ARL9	65	ENST00000479351	110	IPW	155	OLFML2A	200	SNAI1		
21	ARSE	66	ENST00000553247	111	ISOC1	156	OSBPL10	201	SOCS2		
22	ASPN	67	ENST00000567919	112	ITGA8	157	PAX9	202	SOD3		
23	ATOH8	68	ENST00000577614	113	ITGB1BP2	158	PEAR1	203	SPAG16		
24	BAIAP2L2	69	ENST00000590085	114	ITPR1	159	PEG3	204	SPIRE2		
25	C19orf33	70	ENST00000612598	115	KBTBD11	160	PENK	205	SSFA2		
26	C3	71	EYA1	116	KCNJ8	161	PITPNC1	206	ST6GALNAC2		
27	C7orf69	72	FAM107B	117	KCNK12	162	PITX1	207	STAMBPL1		
28	C9orf47	73	FAM162B	118	KDM5D	163	PNMA2	208	STC2		
29	CAMK1D	74	FAM49A	119	KIAA1644	164	PPARG	209	STRA6		
30	CARD16	75	FAM65C	120	KRT32	165	PPP1R1C	210	STXBP6		
31	CARD17	76	FBLN1	121	LAMA3	166	PPP4R4	211	SYNGR3		
32	CCL2	77	FNDC1	122	LAMC2	167	PRDM16	212	TFPI2		
33	CD9	78	FOXF1	123	LINC00578	168	PRSS3	213	TGM2		
34	CDH6	79	FOXF2	124	LINC00640	169	PRSS3P2	214	THBS4		
35	CDO1	80	GAL	125	LINC00842	170	PSG8	215	TLR3		
36	CECR1	81	GBP4	126	LINC00958	171	PTGFRN	216	TMEM176A		
37	CEND1	82	GCA	127	LINC01436	172	PTGIS	217	TMEM30B		
38	CES1	83	GMPR	128	lnc-ADCY9-1	173	PTGS1	218	TMTC1		
39	CHRM2	84	GPAT2	129	lnc-GMDS-2	174	PTK2B	219	TNNT1		
40	CHURC1	85	GPC3	130	lnc-LIFR-1	175	PTN	220	TP53I11		
41	CLDN23	86	GPC4	131	lnc-MYB-1	176	RASGRP3	221	TRIM58		
42	COPS7B	87	GPRC5B	132	LOC100130417	177	RASL11A	222	TSPAN11		
43	CPED1	88	GPX3	133	LOC101927675	178	RASL11B	223	TUBB2B		
44	CXCL1	89	GREM2	134	LOC101929295	179	RDH10	224	UBL4B		
45	CXCL14	90	GSTT2	135	LOC101929398	180	RNF212	225	USP18		

Supplementary Table 2. Upregulated genes with more than 3SDs in PDS2. Gene expression of PDS2 was analyzed by the Agilent microarray method. Comparing with HDF, fold-change, 3SDs Log2(fold-change) were calculated.

Down-regulated genes with more than 3SD in PDS1											
1	A_19_P00803499	31	COL15A1	61	FRMD4A	91	KCNE4	121	LRRC15	151	SKAP2
2	ACAN	32	COL4A5	62	GALNT16	92	KCNG1	122	LRRC17	152	SLC27A5
3	ADAM12	33	CRYBB2	63	GATA2-AS1	93	KCNJ6	123	LYPD6	153	SLC6A9
4	ADAMTSL1	34	CRYBB2P1	64	GFRA1	94	KCNK1	124	LYPD6B	154	SLC7A5
5	ADH1A	35	CXCL12	65	GLS	95	KCNK2	125	LZTS1	155	SPP1
6	ADH1C	36	DC378344	66	GNG4	96	KIF26B	126	MALL	156	SYPL2
7	ADM2	37	DNAH12	67	GPR162	97	KRT18	127	MAP2K6	157	SYT1
8	ADRA1D	38	DPEP3	68	GRP	98	KRT18P55	128	MEOX2	158	SYTL2
9	ADRA2A	39	DPT	69	GRPR	99	KRT19	129	MEST	159	TBX1
10	AGT	40	EGR2	70	HAPLN1	100	KRT19P2	130	MEX3B	160	TENM2
11	AMIGO2	41	ELTD1	71	HAS1	101	LFNG	131	MOCS3	161	TES
12	ANXA2R	42	EMB	72	HECTD2	102	LIMCH1	132	MYOD1	162	TGFB3
13	APCDD1L	43	EMX2OS	73	HEPH	103	LINC00673	133	NETO2	163	THC2596076
14	AQP1	44	ENST00000382641	74	HIVEP3	104	LINC01013	134	NNMT	164	TINAGL1
15	ATP7B	45	ENST00000430381	75	HMCN1	105	LINC01116	135	NTF3	165	TMEM217
16	BAALC	46	ENST00000432694	76	HMSD	106	LINC01119	136	NXPH4	166	TNFSF4
17	BACE2	47	ENST00000438158	77	HNF4A-AS1	107	LINC01133	137	PCBP3	167	TNFSF9
18	BHLHE40	48	ENST00000520524	78	HOTAIRM1	108	LIPC	138	PCOLCE2	168	TPD52L1
19	BRINP1	49	ENST00000619960	79	HOXA10-AS	109	lnc-ADA-1	139	PDE11A	169	ULBP1
20	C10orf10	50	EPB41L3	80	HOXA13	110	lnc-ATIC-2	140	PDE1A	170	UNC5B
21	C11orf96	51	ERAP2	81	HOXB-AS3	111	lnc-CCDC69-1	141	PDK3	171	WISP2
22	CA12	52	FADS2	82	HOXC4	112	lnc-EBF1-2	142	PLA2G4C	172	WNK4
23	CARD9	53	FAM19A5	83	HOXC6	113	lnc-FANK1-3	143	PLAC8	173	WNT2
24	CCDC8	54	FAM20A	84	HOXD-AS2	114	lnc-IRX3-4	144	PPP1R13L	174	XLOC_l2_011265
25	CDCA7	55	FBXO32	85	IGF2BP1	115	lnc-SCRG1-1	145	PRAME	175	XLOC_l2_014835
26	CGNL1	56	FIBCD1	86	IL20RB	116	lnc-YIF1A-6	146	RBMS3-AS3	176	XLOC_l2_015849
27	CH25H	57	FIBIN	87	IRX2	117	LOC100130938	147	REP15		
28	CMKLR1	58	FOS	88	ITGA11	118	LOC100507420	148	SCD		
29	CMTR1	59	FOXL2	89	ITGA9	119	LOC401557	149	SGCA		
30	COL12A1	60	FOXL2NB	90	ITGB2	120	LOXL4	150	SHROOM3		

Supplementary Table 3. Downregulated genes with more than 3SDs in PDS1. Gene expression of PDS1 was analyzed by the Agilent microarray method. Comparing with HDF, fold-change, 3SDs Log2(fold-change) were calculated.

Down-regulated genes with more than 3SD in PDS2											
1	A_19_P00803499	36	DPP9-AS1	71	HAS2	106	lnc-DEC1-3	141	NOTCH3	176	TM4SF1
2	A_33_P3255824	37	EDIL3	72	HEPH	107	lnc-FANK1-3	142	NTF3	177	TMEM158
3	ACKR3	38	EFHD1	73	HIVEP3	108	lnc-FBXO25-3	143	NXPH4	178	TMEM217
4	ADAM12	39	EFNB2	74	HMCN1	109	lnc-FOXL1-2	144	OSBPL6	179	TMEM255B
5	ADAMTSL1	40	EGR2	75	HMSD	110	lnc-IRX3-4	145	OSR2	180	TNFSF4
6	ADRA2A	41	ELTD1	76	HOTAIRM1	111	lnc-RP11-597K23.2.1-2	146	PADI1	181	TNFSF9
7	AGT	42	EMX2OS	77	HOXA10-AS	112	lnc-YIF1A-6	147	PAPPA	182	TPD52L1
8	AIM1	43	ENPP2	78	HOXA5	113	LOC100506947	148	PAQR5	183	UNC5B
9	AK5	44	ENST00000382641	79	HOXB2	114	LOC100507420	149	PAX8-AS1	184	VEPH1
10	AMIGO2	45	ENST00000430381	80	HOXB-AS1	115	LOC101928880	150	PCOLCE2	185	WASF3
11	ANKRD20A12P	46	ENST00000520819	81	HOXB-AS3	116	LOC101929484	151	PDE1C	186	WISP2
12	ANXA2R	47	ENST00000607314	82	HOXC9	117	LOC400043	152	PEG10	187	WNK4
13	AOX1	48	ERAP2	83	HOXD9	118	LOC729080	153	PIEZO2	188	ZFHX4
14	APCDD1L	49	EXTL1	84	IFITM10	119	LOXL1	154	PLA2G4C	189	ZNF385D
15	APCDD1L-AS1	50	FAM19A5	85	IGF1	120	LOXL4	155	PLXDC2		
16	AQP1	51	FAM20A	86	IL20RB	121	LRRC15	156	PNPLA7		
17	ARL4C	52	FAM84A	87	INHBB	122	LRRC32	157	PODXL		
18	BAALC	53	FBXO32	88	INPP4B	123	LRRN4CL	158	POSTN		
19	BACE2	54	FGF13	89	ITGA11	124	LUM	159	PTGDS		
20	BCL11A	55	FIBCD1	90	ITGA7	125	MALL	160	QPCT		
21	BCL7A	56	FIBIN	91	KANK4	126	MAP2K6	161	RASGRP1		
22	C11orf96	57	FOLR3	92	KCNE4	127	MAP3K7CL	162	SCAMP5		
23	CA13	58	FOXL1	93	KCNK2	128	MDGA1	163	SCIN		
24	CCDC85A	59	FRAS1	94	KIAA1549L	129	MEDAG	164	SGCA		
25	CD24	60	G0S2	95	KLHL30	130	MEIS1	165	SIM1		
26	CDA	61	GABBR2	96	LFNG	131	MEX3B	166	SLC1A3		
27	CDCP1	62	GALNT16	97	LINC00607	132	MOXD1	167	SLIT2		
28	CFD	63	GATA2-AS1	98	LINC00673	133	MXRA5	168	SP6		
29	CH25H	64	GCNT1	99	LINC00942	134	MYBL1	169	STEAP3		
30	CLEC3B	65	GGT5	100	LINC01013	135	NALCN	170	SUSD2		
31	CLN8	66	GPC6	101	LINC01116	136	NDUFA4L2	171	SYNPO2L		
32	CMTR1	67	GPM6B	102	LINC01119	137	NEK7	172	SYPL2		
33	CNIH3	68	GRIA1	103	LINC01540	138	NETO2	173	TBX1		
34	CORO2B	69	GRP	104	LIPC	139	NGEF	174	TENM2		
35	CPEB1	70	HAPLN1	105	lnc-ANKRD20A1-2	140	NLGN1	175	THBD		

Supplementary Table 4. Downregulated genes with more than 3SDs in PDS2. Gene expression of PDS2 was analyzed by the Agilent microarray method. Comparing with HDF, fold-change, 3SDs Log2(fold-change) were calculated.

Types of cells	Recruited BMDCs and subsets			
	**HSC-2 alone**	**HSC-2+HDF**	**HSC-2+PDS1**	**HSC-2+PDS2**
BMDCs (GFP-positive)	Low	High*	High****	High***
CAFs (αSMA-positive)	n.d	n.d	n.d	n.d
TECs (CD34-positive)	n.d	n.d	n.d	n.d
TAMs (CD11b-positive)	High	Low*	Low	Low
MDSCs (GR1/CD11b/Arg1 triple-positive)	Low	Low	$^{\Phi}$ High****	$^{\Phi}$ High****

Supplementary Table 5. Summarizing the data of BMDC infiltration and subsets in OSCC tumor models. Significant difference (* $P < 0.05$, *** $P < 0.001$, **** $P < 0.0001$) presented between the control group (HSC-2) and tumor/stroma co-xenografted groups (HSC-2+HDF, HSC-2+PDS1, and HSC-2+PDS2). n.d., not differentiated. $^{\Phi}$, significant difference ($P < 0.0001$) presented between HSC-2+PDS1/2 and HSC-2+HDF.

Primary antibody	Immunized animal	Antigen retrieval	Dilution	Supplier
GFP	Goat	Microwave heating in 0.01 mol/L citrate buffer (pH 6.0) at 100°C for 1 min	1:500	Abcam (ab6673)
αSMA	Rabbit	Microwave heating in 0.01 mol/L citrate buffer (pH 6.0) at 100°C for 1 min	1:200	Abcam (ab7817)
CD34	Rabbit	Microwave heating in 0.01 mol/L citrate buffer (pH 6.0) at 100°C for 1 min	1:200	Abcam (ab81289)
CD11b	Rabbit	Microwave heating in 0.01 mol/L citrate buffer (pH 6.0) at 100°C for 1 min	1:500	Abcam (ab133357)
GR1	Rat	0.1% Trypsin at 37°C for 5 min	1:200	Biolegend (#108401)
Arginase 1 (Arg1)	Rabbit	Microwave heating in 0.01 mol/L citrate buffer (pH 6.0) at 100°C for 1 min	1:100	Cell signaling technology (#93668)
CCL2	Rabbit	Microwave heating in 0.01 mol/L citrate buffer (pH 6.0) at 100°C for 1 min	1:500	Abcam (ab25124)
CCR2	Rabbit	Microwave heating in 0.01 mol/L Tris-EDTA buffer (pH 9.0) at 100°C for 1 min	1:250	Abcam (ab273050)
SDF-1	Rabbit	Microwave heating in 0.01 mol/L citrate buffer (pH 6.0) at 100°C for 1 min	1:200	Abcam (ab9797)
HGF	Rabbit	Cooker heating in 0.01 mol/L Tris-EDTA buffer (pH 9.0) at 100°C for 8 min	1:200	Abcam (ab83760)
cMet	Rabbit	Cooker heating in 0.01 mol/L citrate buffer (pH 6.0) at 100°C for 8 min	1:200	Abcam (ab51067)
CXCR4	Rabbit	Cooker heating in 0.01 mol/L citrate buffer (pH 6.0) at 100°C for 8 min	1:300	Abcam (ab124824)

Supplementary Table 6. Antibodies used in immunohistochemistry

Secondary antibody	Immunized animal	Fluorescent dye	Supplier
Anti-goat IgG	Donkey	Alexa Fluor 488	Thermo (Ref: A11055)
Anti-rabbit IgG	Donkey	Alexa Fluor 568	Thermo (Ref: A10042)

Supplementary Table 7. Antibodies used in double-fluorescent immunohistochemistry
